# Supplementary material for: Fast light-switchable polymeric carbon nitride membranes for tunable gas separation
Source: Nat Commun. 2022 Nov 26;13:7299. doi: 10.1038/s41467-022-35013-x (PMC9701225; doi:10.1038/s41467-022-35013-x)
Supplement: Supplementary file 1 — Supplementary Information [file 41467_2022_35013_MOESM1_ESM.pdf]

Supplementary Information for

Fast light-switchable polymeric carbon nitride membranes for tunable gas separation

Timur Ashirov, Julya Stein Siena, Mengru Zhang, A. Ozgur Yazaydin, Markus Antonietti, Ali Coskun**

Supplementary Figures

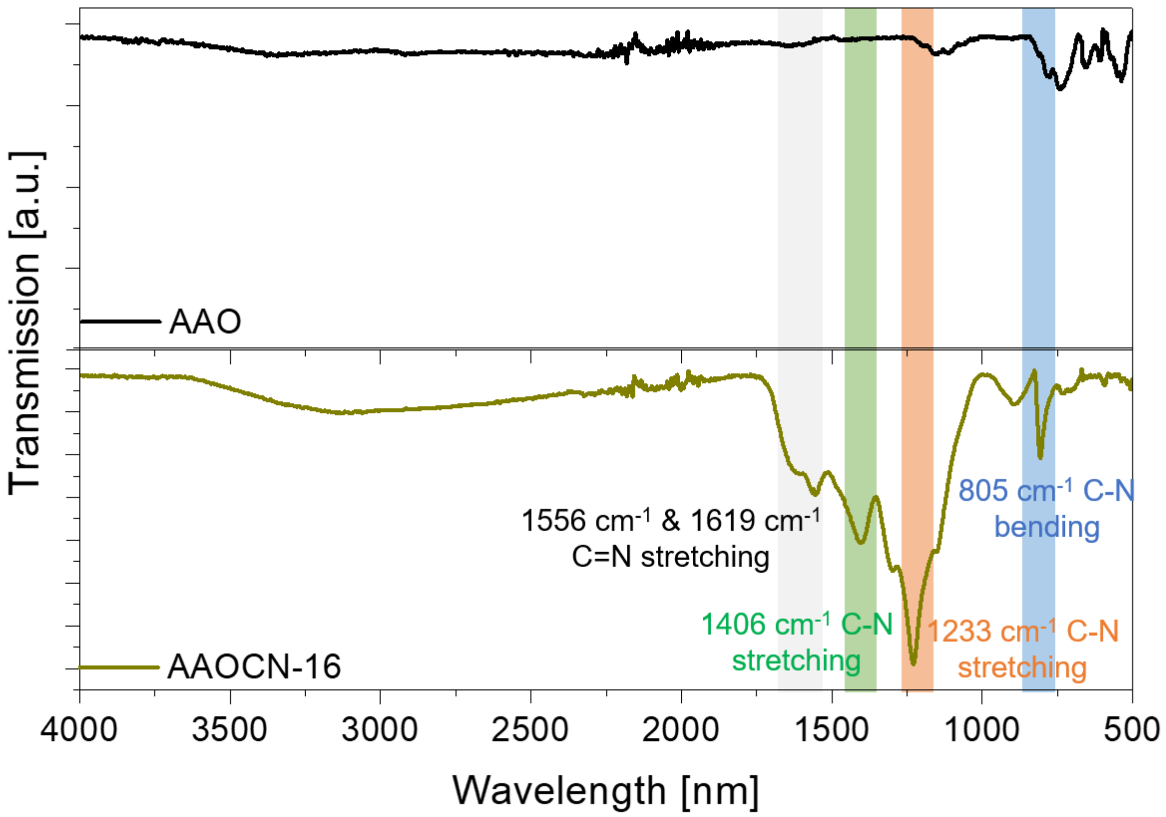

Supplementary Figure 1. FTIR spectra of blank AAO and AAOCN-16 sample. The characteristic -C-N-, -C=N- vibration bands of polymeric carbon nitride can be clearly observed in the FTIR spectra of AAOCN-16 sample.

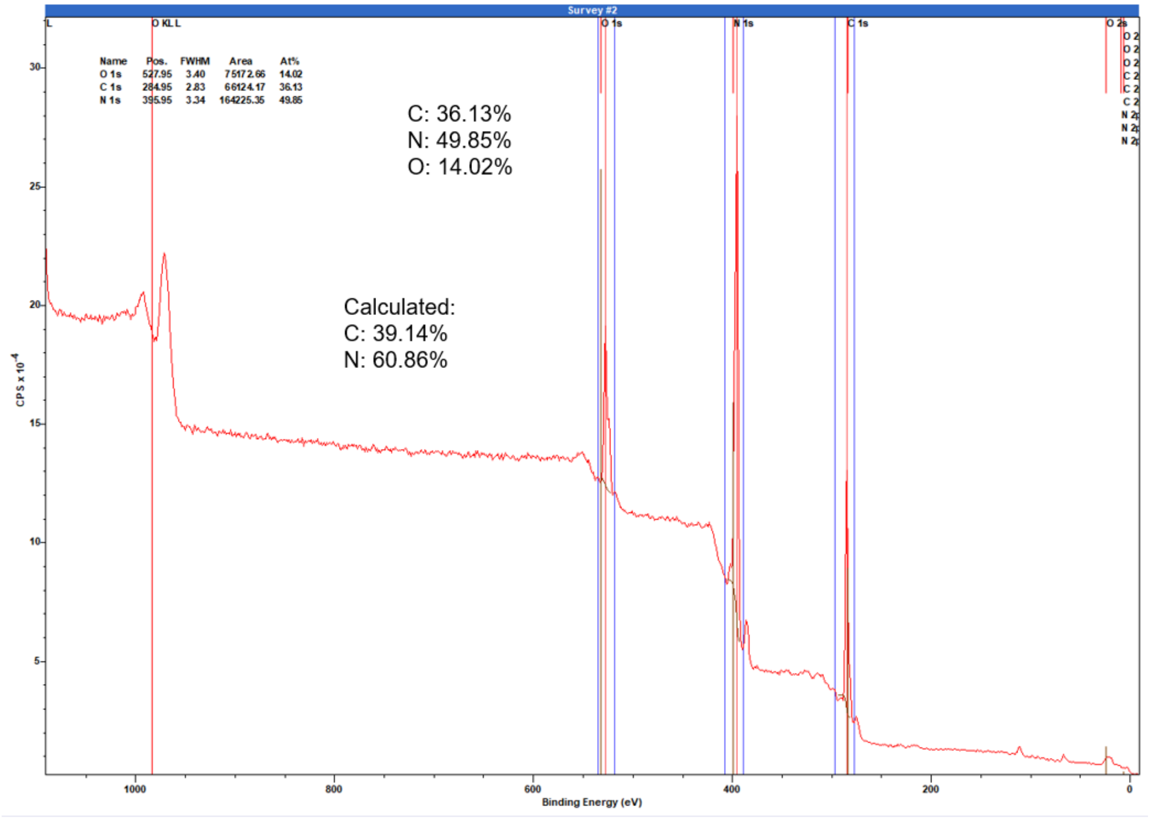

Supplementary Figure 2. XPS survey spectra of AAOCN-16 sample. The XPS spectra revealed near ideal content of carbon and lower amount of nitrogen indicating presence of defects.

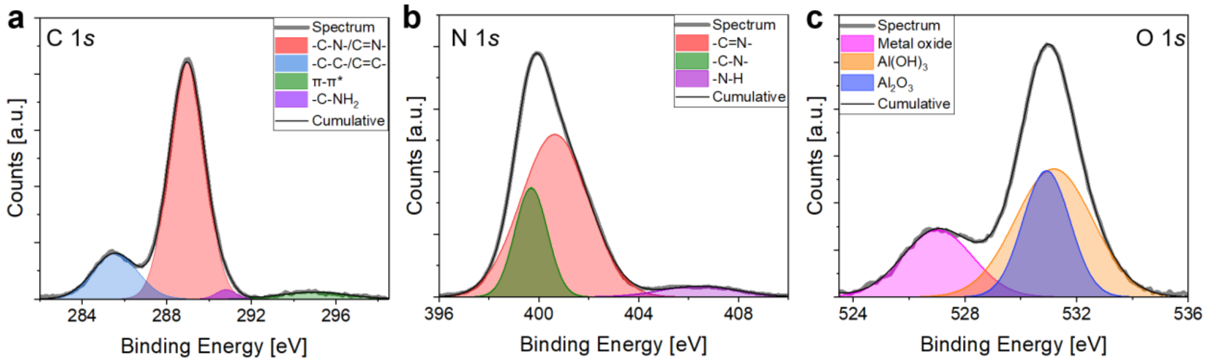

Supplementary Figure 3. High resolution (a) C 1*s*, (b) N 1*s* and (c) O 1*s* spectra of AAOCN-16 membrane. High resolution C 1*s* and N 1*s* spectra revealed typical –C-N-/-C=N- moieties. Peaks observed in high resolution O 1*s* spectra were attributed to AAO.

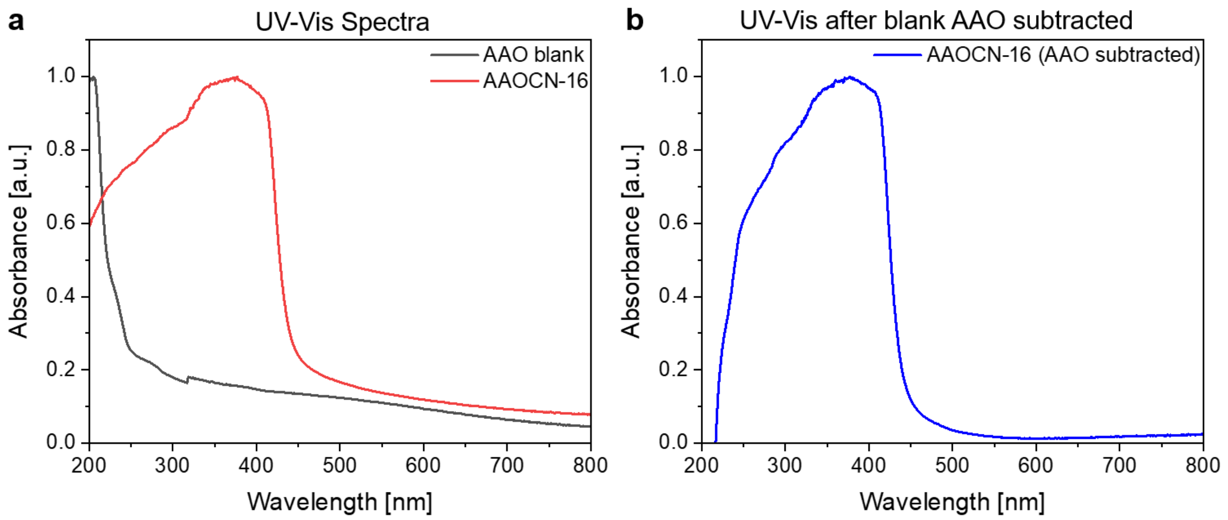

Supplementary Figure 4. The UV-Vis spectra of (a) AAOCN-16 and AAO blank. (b) AAOCN-16 after subtraction of AAO blank spectra.

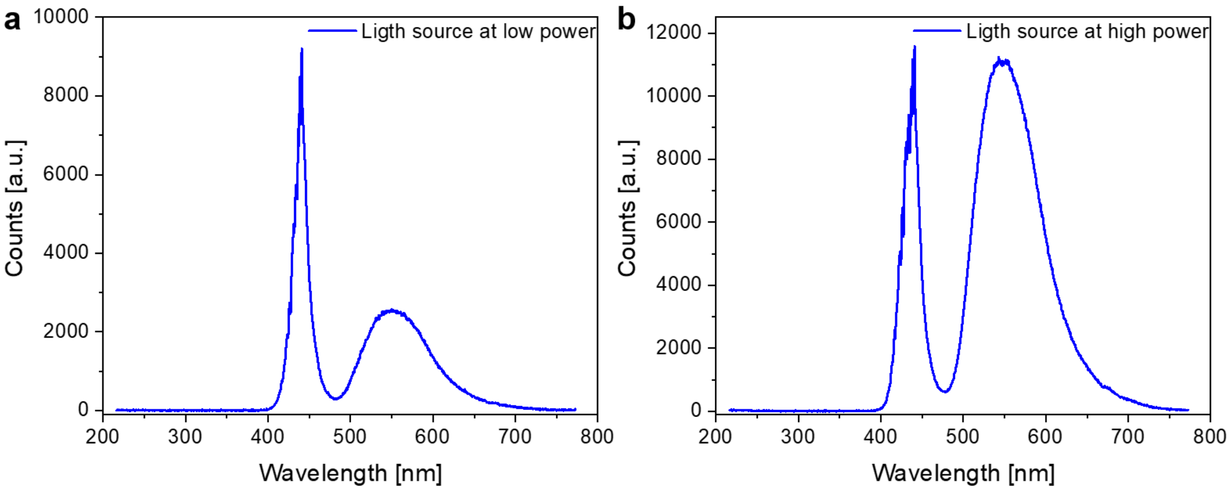

Supplementary Figure 5. The emission spectra of LED source (a) at low and (b) at high power levels.

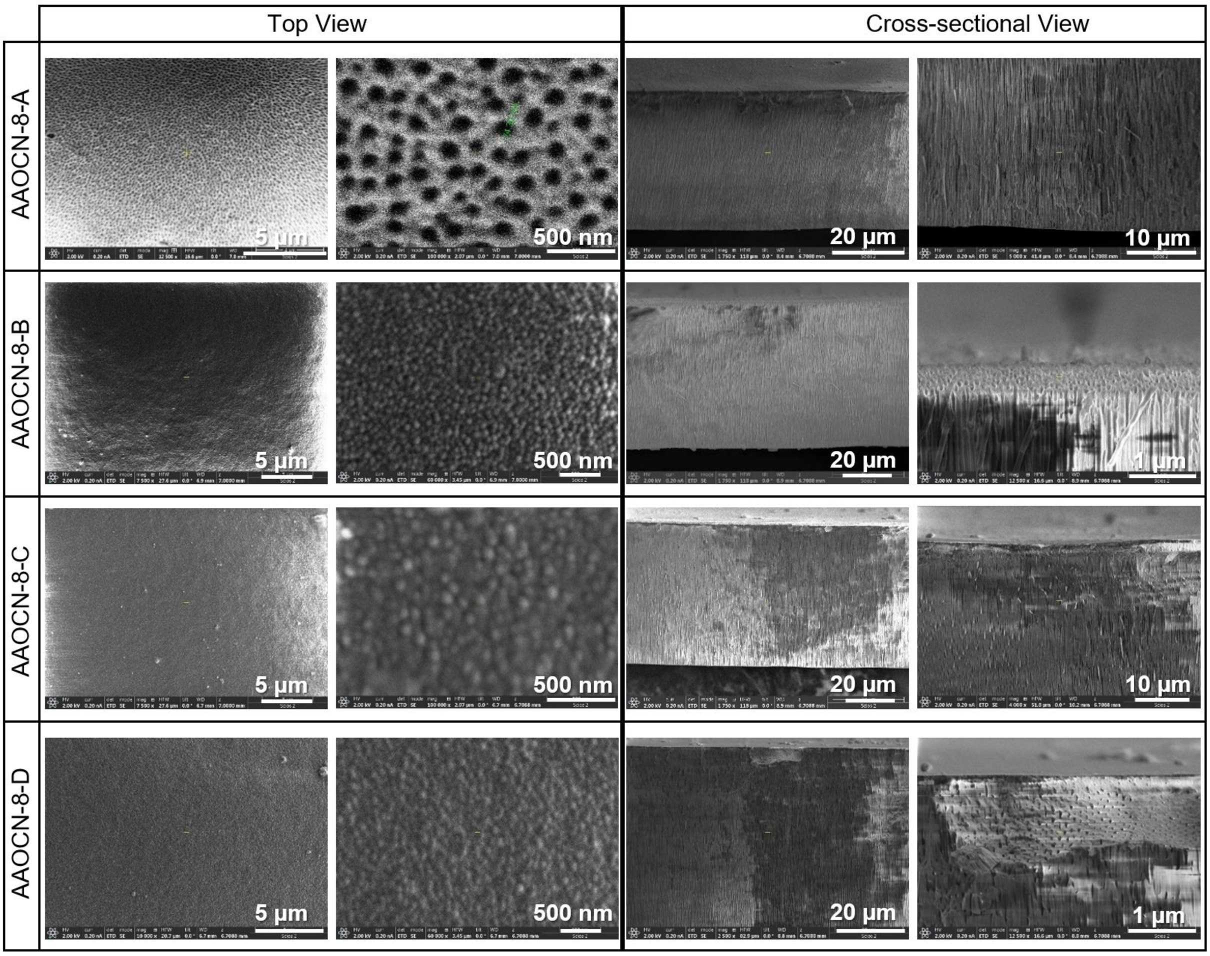

Supplementary Figure 6. Top and cross-sectional view SEM images of AAOCN-8 samples positioned at different distances from the melamine source in LPCVD with A being the closest and D being the furthest. As it can be seen from the SEM images, placing the AAO further from the melamine source blocks the pores completely.

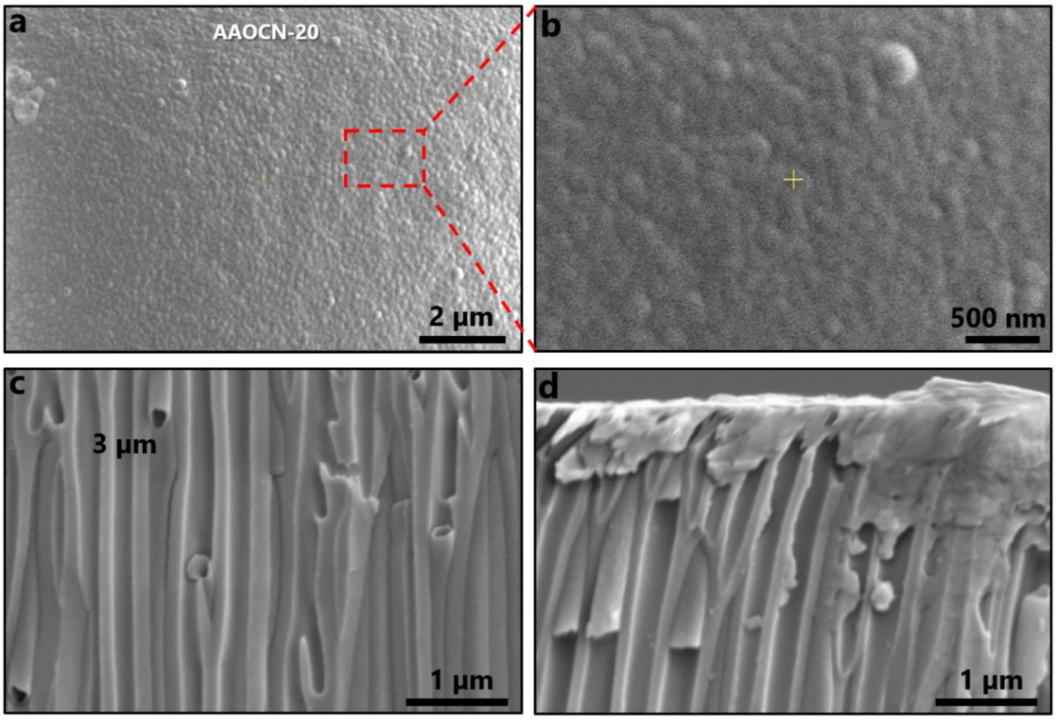

Supplementary Figure 7. (a) Top view, (b) Zoomed-in regional and (c-d) Cross-sectional view SEM images of AAOCN-20 sample. Both from the top-view and the cross-sectional view SEM images, pore blockage was clearly observed.

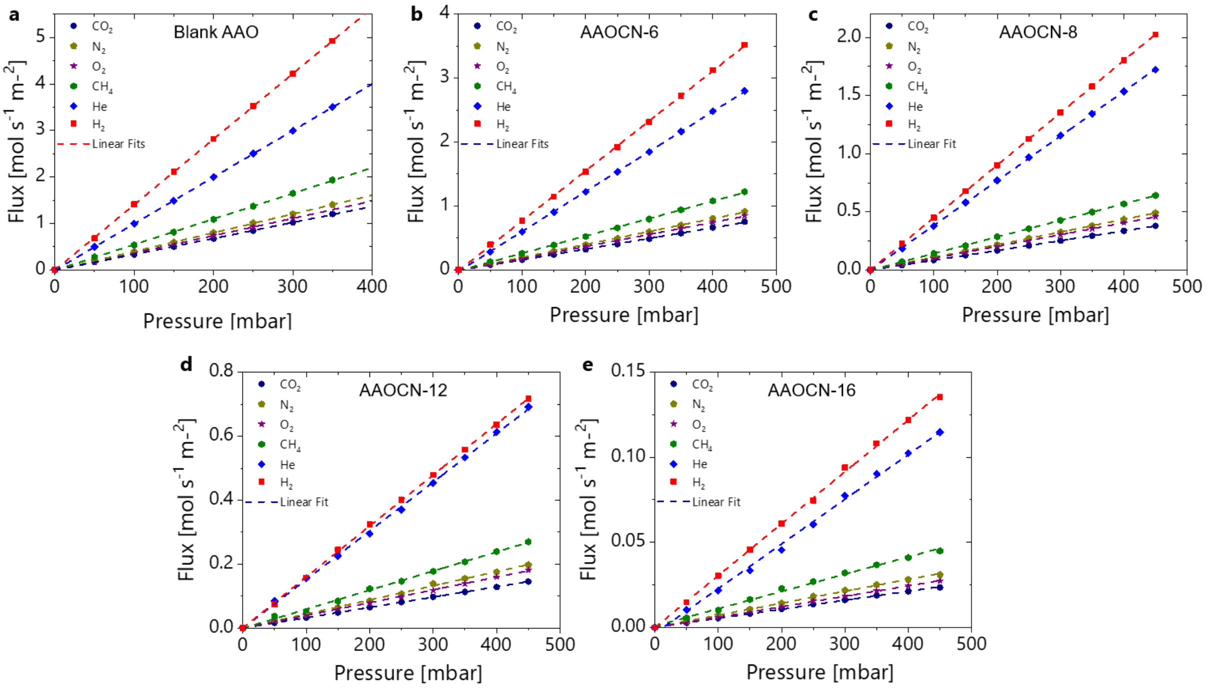

Supplementary Figure 8. Flux vs pressure curves of (a) blank AAO, (b) AAOCN-6, (c) AAOCN-8, (d) AAOCN-12 and (e) AAOCN-16 samples for H_2, He, CH_4, N_2, O_2 and CO_2. The deposition of pCN leads to a decrease in the permeance due to decreasing pore size.

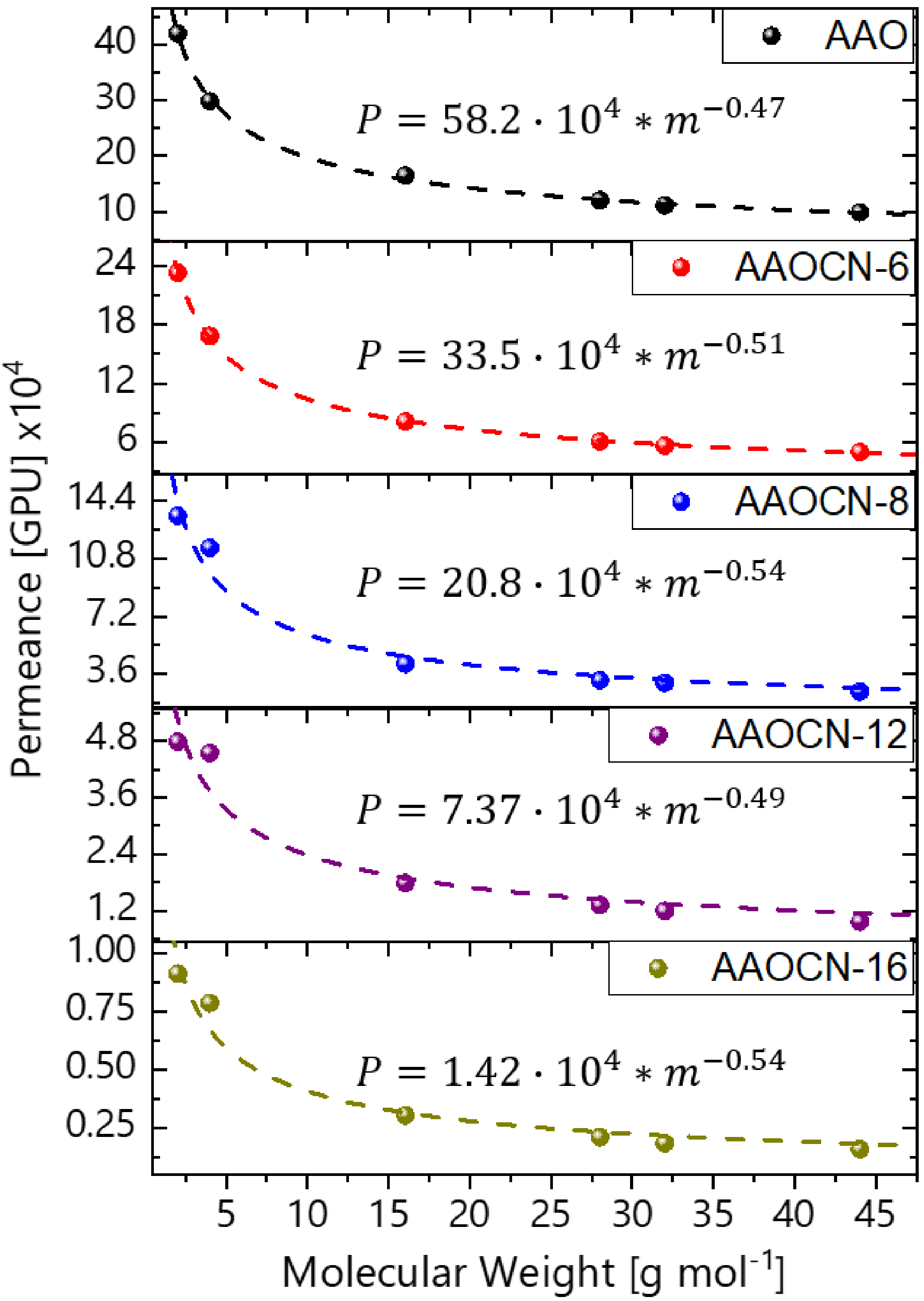

Supplementary Figure 9. The plots of permeance vs molecular weight of gases for blank AAO and AAOCN membranes. The inset equations show the fitting of the data points according to the Knudsen model.

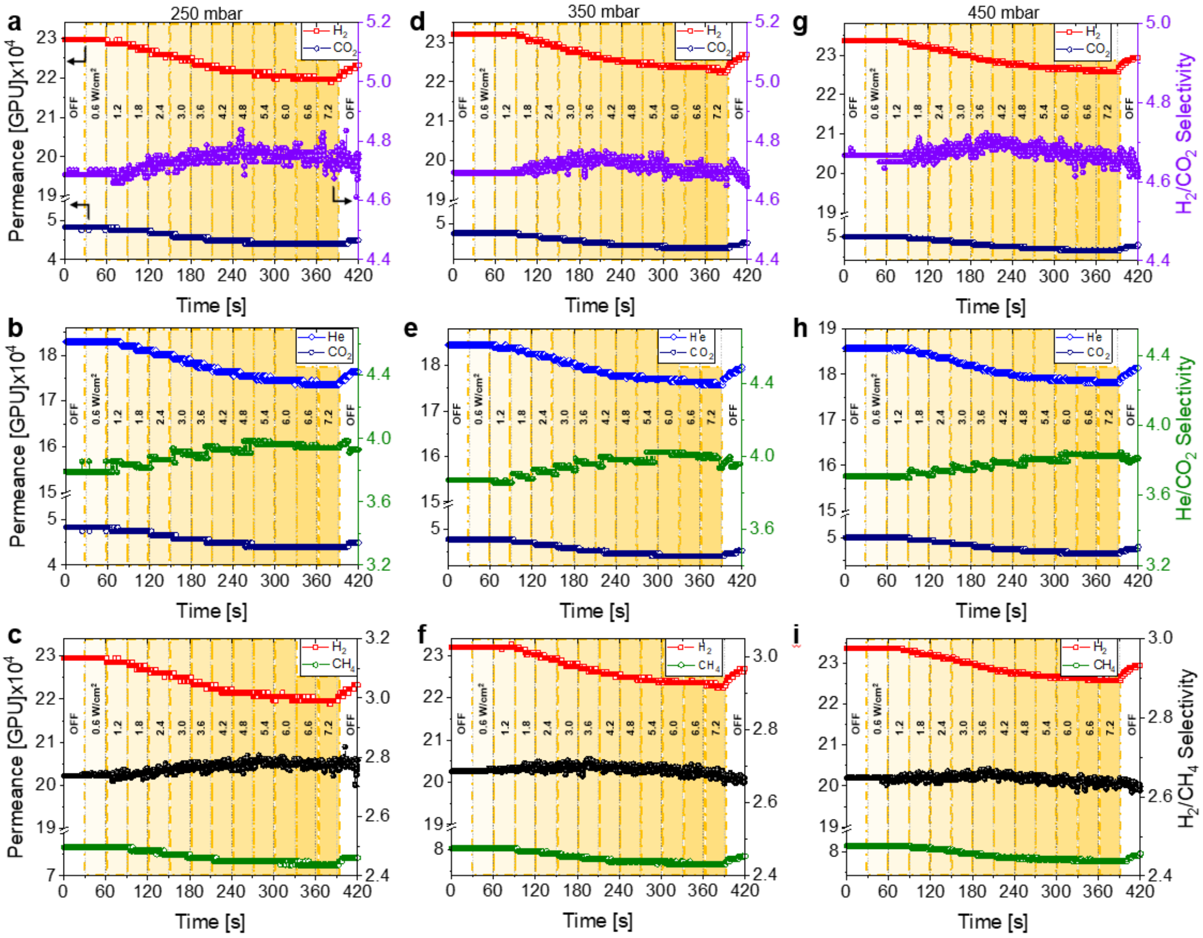

Supplementary Figure 10. The change of gas permeance and selectivity vs light intensity (W/cm^2) on AAOCN-6 for H_2/CO_2, He/CO_2 and H_2/CH_4 separation at 250 mbar (a), (b), (c), at 350 mbar (d), (e), (f), and at 450 mbar (g), (h), (i) transmembrane pressure, respectively. The light intensity (W/cm^2) is shown in the middle and increases from left to right in accordance with the intensity of yellow background.

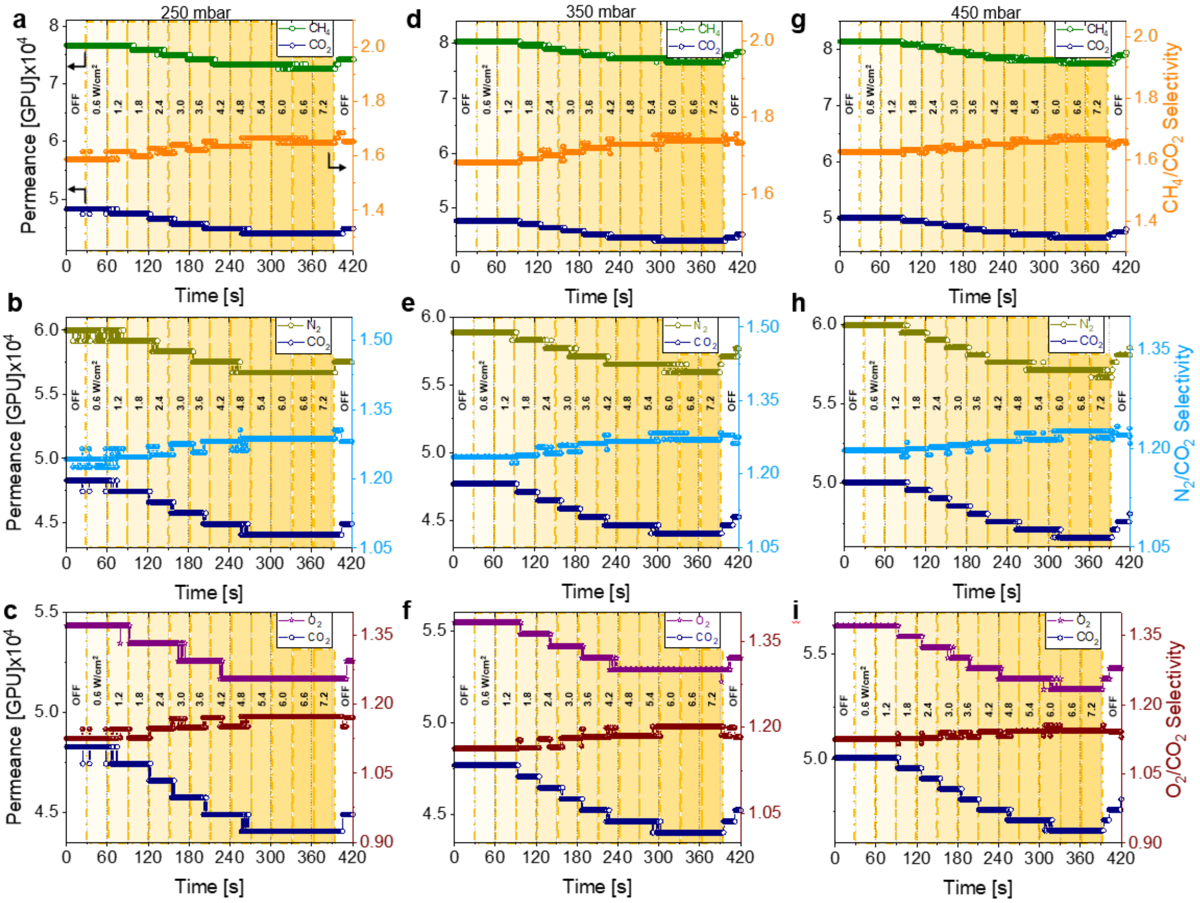

Supplementary Figure 11. The change of gas permeance and selectivity vs light intensity (W/cm^2) on AAOCN-6 for CH_4/CO_2, N_2/CO_2 and O_2/CO_2 at 250 mbar (a), (b), (c), at 350 mbar (d), (e), (f), and at 450 mbar (g), (h), (i) transmembrane pressure, respectively. The light intensity (W/cm^2) is shown in the middle and increases from left to right in accordance with the intensity of yellow background.

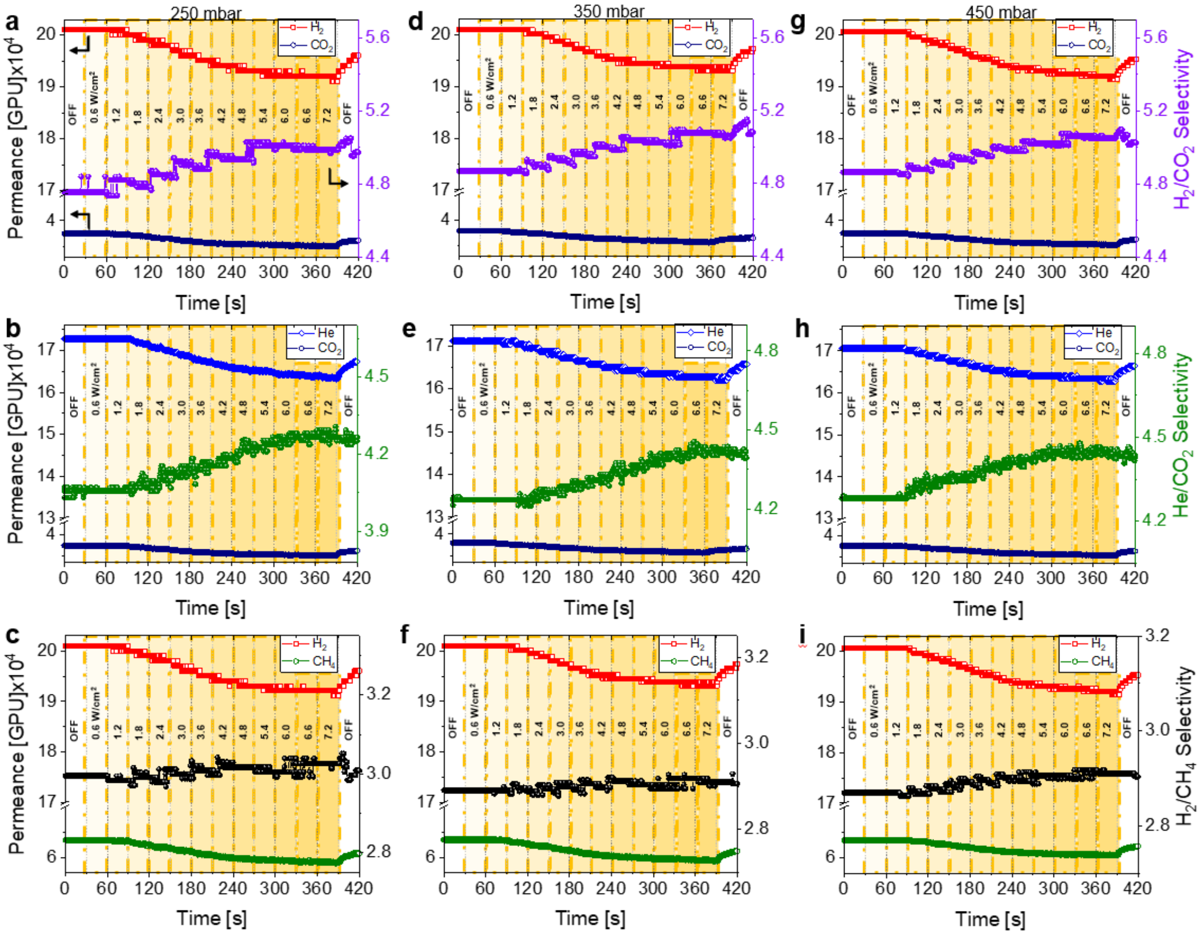

Supplementary Figure 12. The change of gas permeance and selectivity vs light intensity (W/cm^2) on AAOCN-8 for H_2/CO_2, He/CO_2 and H_2/CH_4 at 250 mbar (a), (b), (c), at 350 mbar (d), (e), (f), and at 450 mbar (g), (h), (i) transmembrane pressure, respectively. The light intensity (W/cm^2) is shown in the middle and increases from left to right in accordance with the intensity of yellow background.

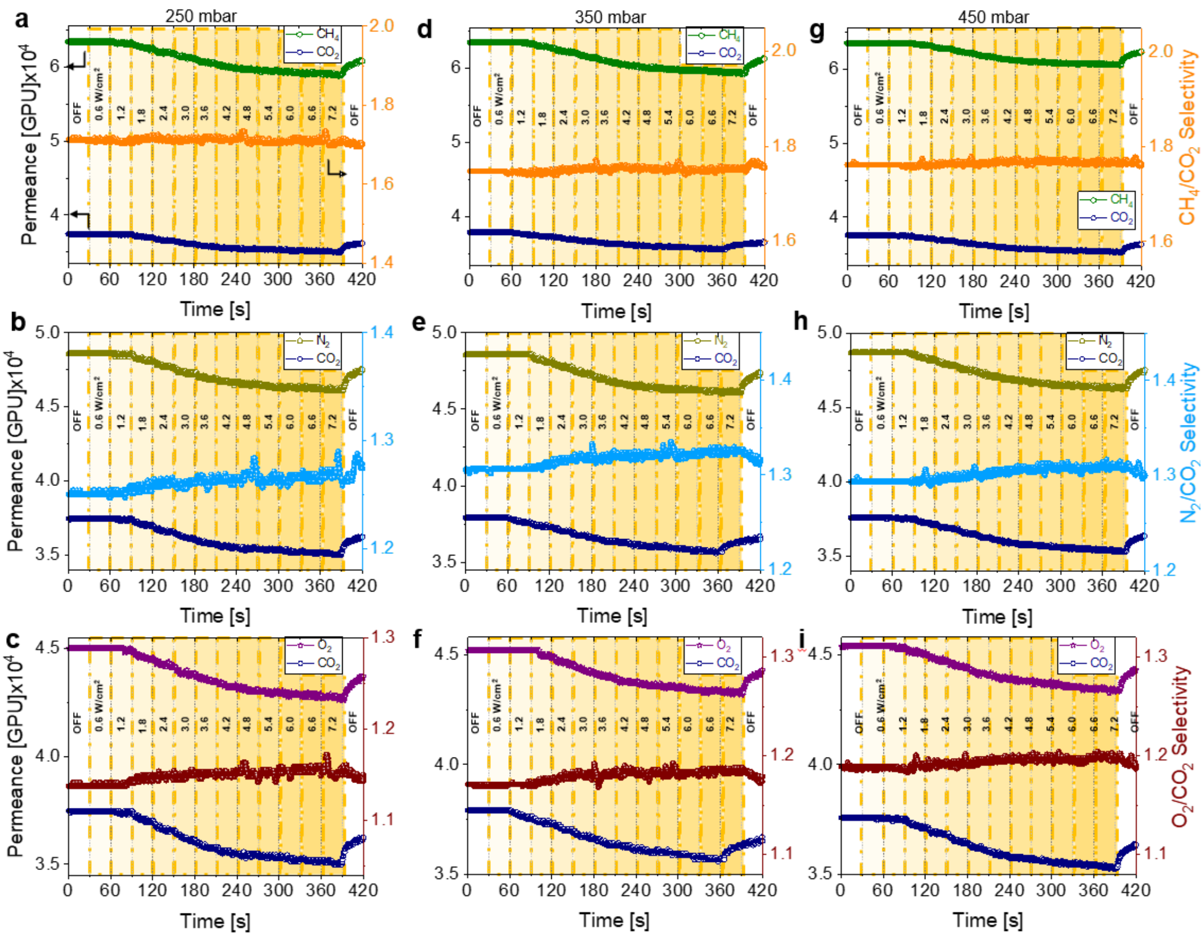

Supplementary Figure 13. The change of gas permeance and selectivity vs light intensity (W/cm^2) on AAOCN-8 for CH_4/CO_2, N_2/CO_2 and O_2/CO_2 at 250 mbar (a), (b), (c), at 350 mbar (d), (e), (f), and at 450 mbar (g), (h), (i) transmembrane pressure, respectively. The light intensity (W/cm^2) is shown in the middle and increases from left to right in accordance with the intensity of yellow background.

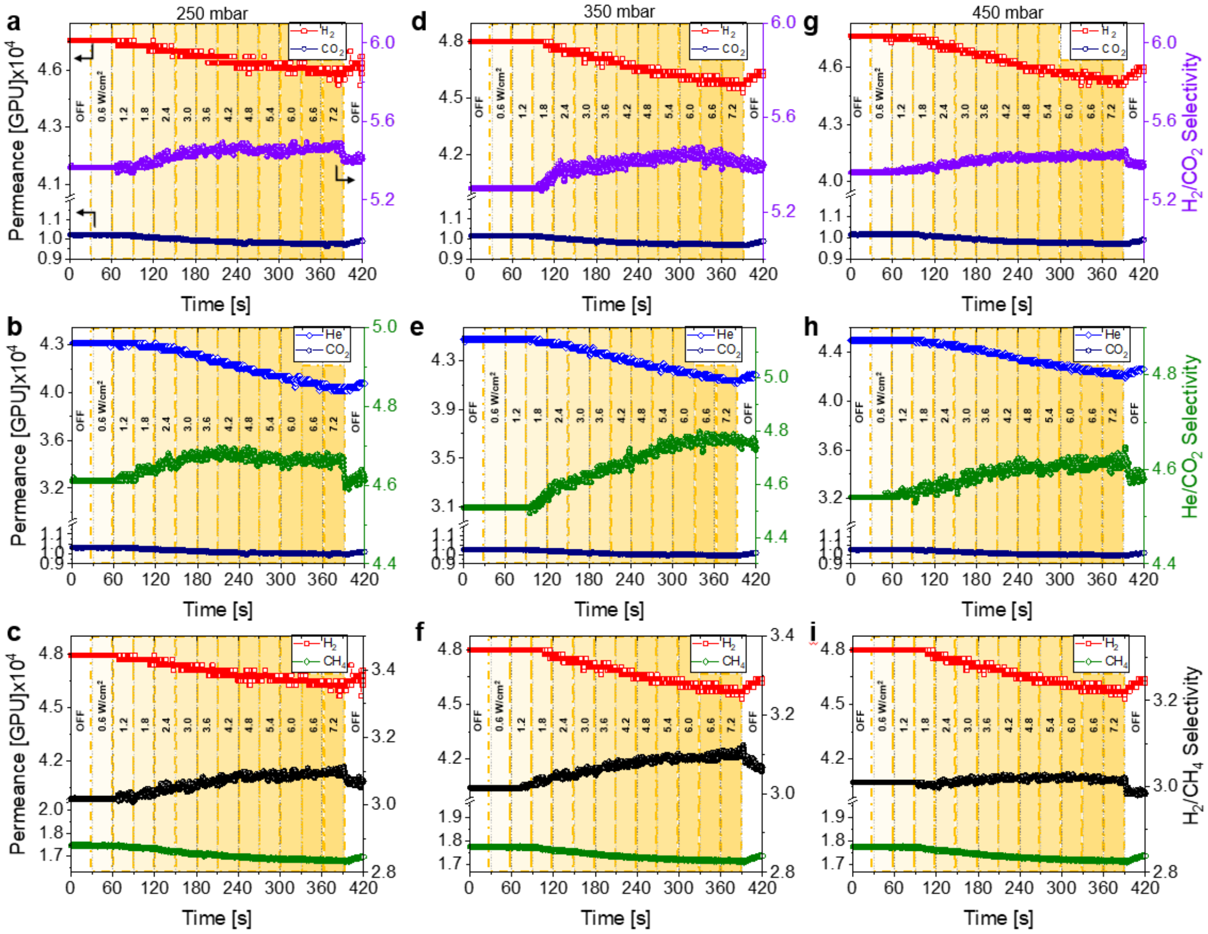

Supplementary Figure 14. The change of gas permeance and selectivity vs light intensity (W/cm^2) on AAOCN-12 for H_2/CO_2, He/CO_2 and H_2/CH_4 at 250 mbar (a), (b), (c), at 350 mbar (d), (e), (f), and at 450 mbar (g), (h), (i) transmembrane pressure, respectively. The light intensity (W/cm^2) is shown in the middle and increases from left to right in accordance with the intensity of yellow background.

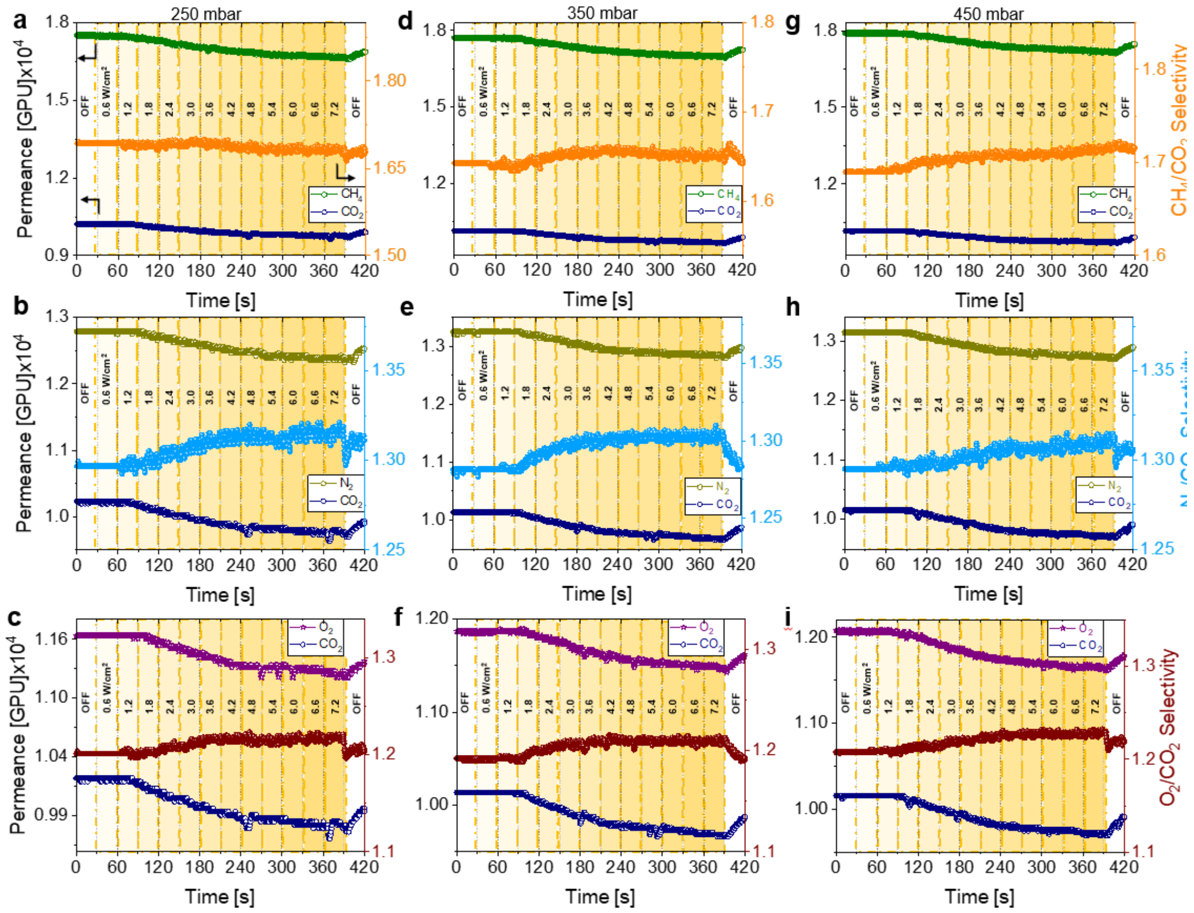

Supplementary Figure 15. The change of gas permeance and selectivity vs light intensity (W/cm^2) on AAOCN-12 for CH_4/CO_2, N_2/CO_2 and O_2/CO_2 at 250 mbar (a), (b), (c), at 350 mbar (d), (e), (f), and at 450 mbar (g), (h), (i) transmembrane pressure, respectively. The light intensity (W/cm^2) is shown in the middle and increases from left to right in accordance with the intensity of yellow background.

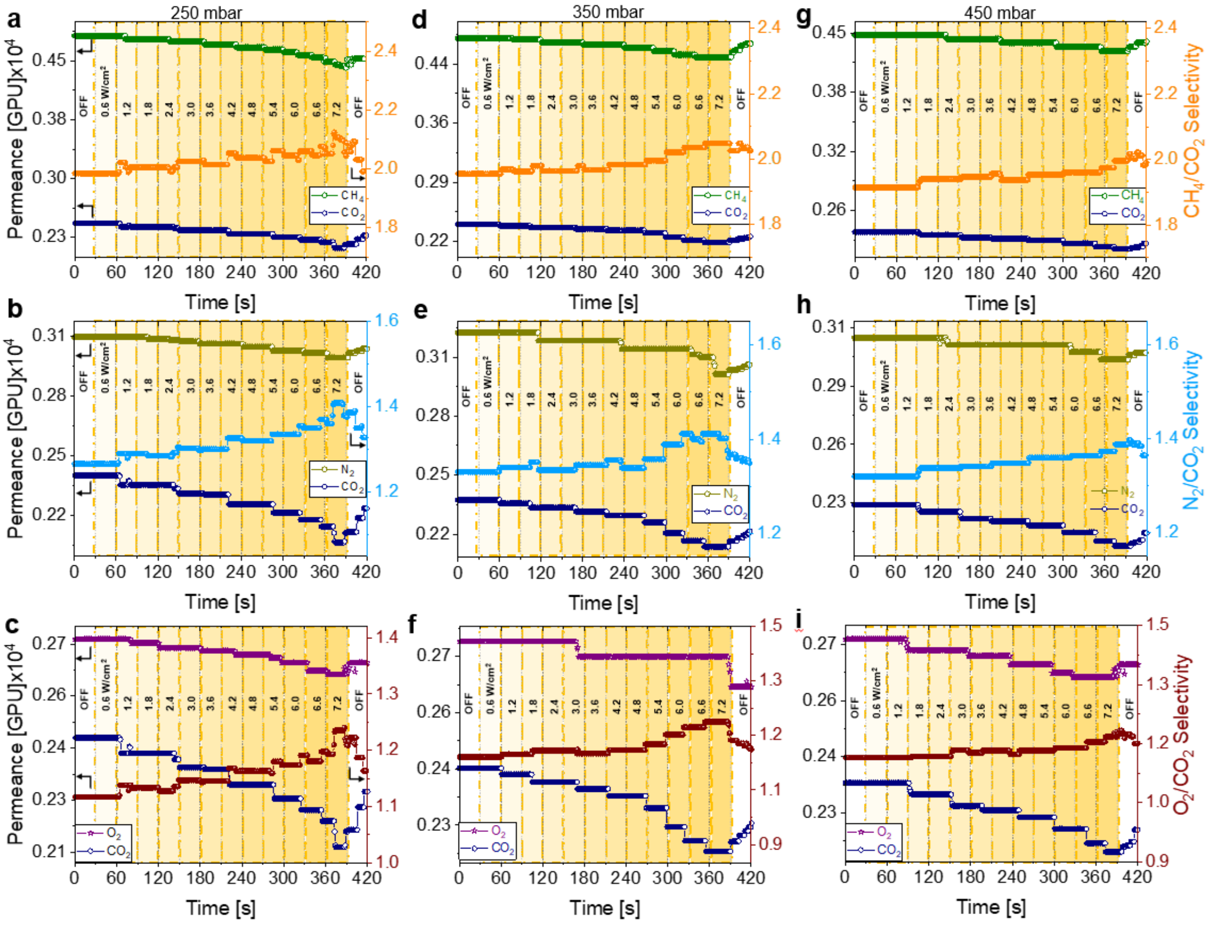

Supplementary Figure 16. The change of gas permeance and selectivity vs light intensity (W/cm^2) on AAOCN-16 for CH_4/CO_2, N_2/CO_2 and O_2/CO_2 at 250 mbar (a), (b), (c), at 350 mbar (d), (e), (f), and at 450 mbar (g), (h), (i) transmembrane pressure, respectively. The light intensity (W/cm^2) is shown in the middle and increases from left to right in accordance with the intensity of yellow background.

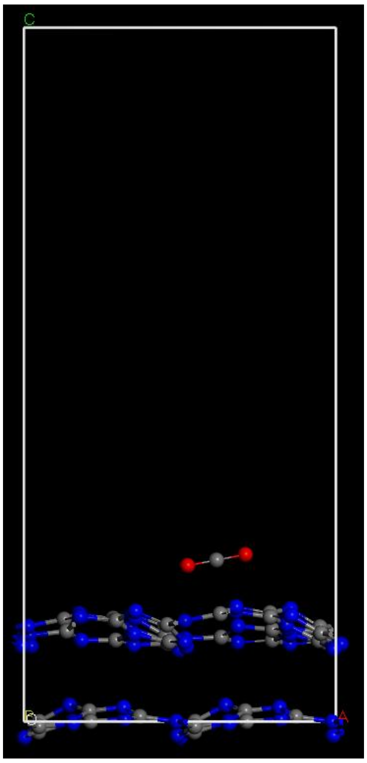

Supplementary Figure 17. A simulation box that illustrates the periodic DFT optimized position of CO_2 molecule over two layers of g-C_3N_4.

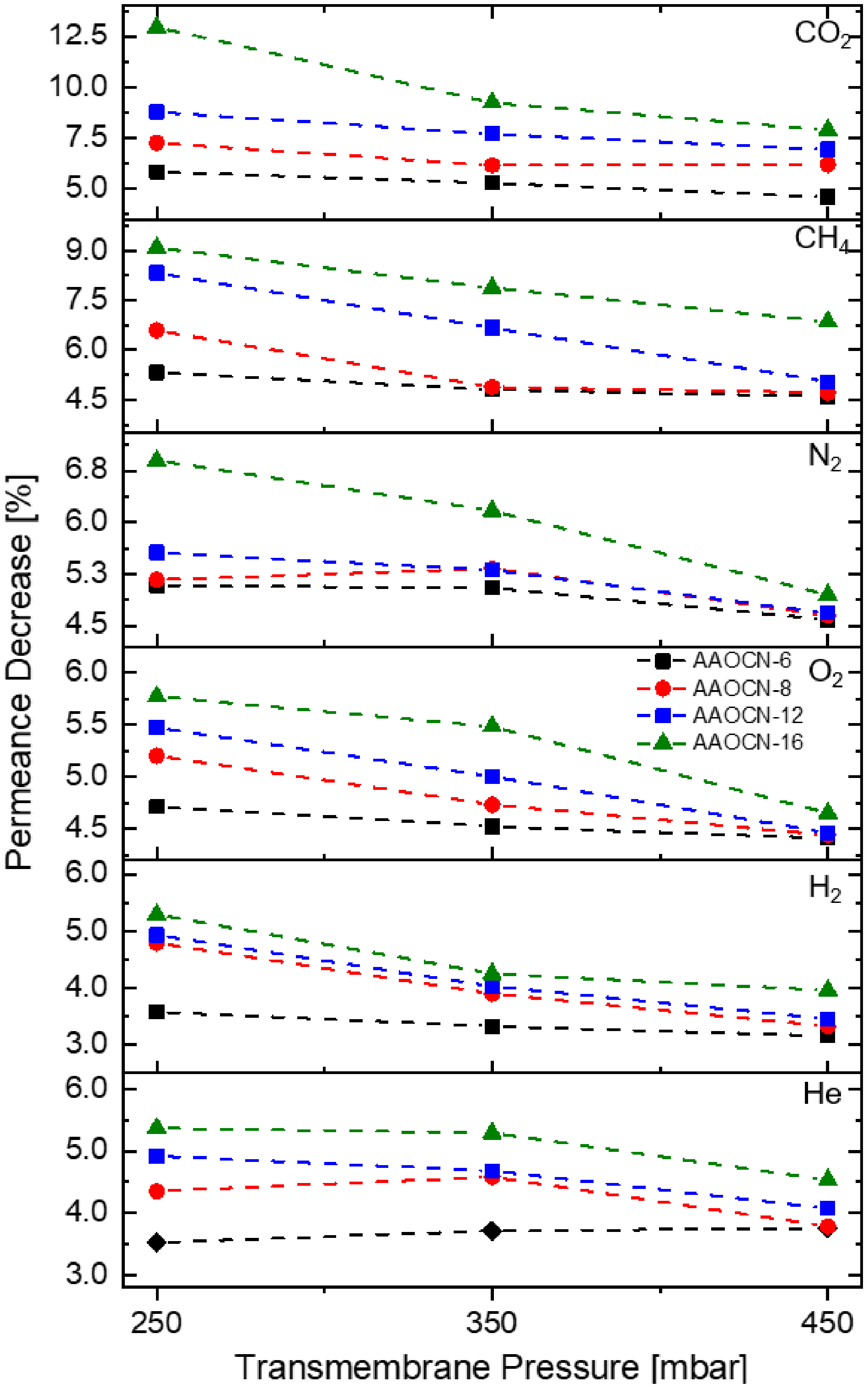

Supplementary Figure 18. The decrease in gas permeance at maximum light intensity power (7.2 W/cm^2) vs transmembrane pressure for **AAOCN-X** samples.

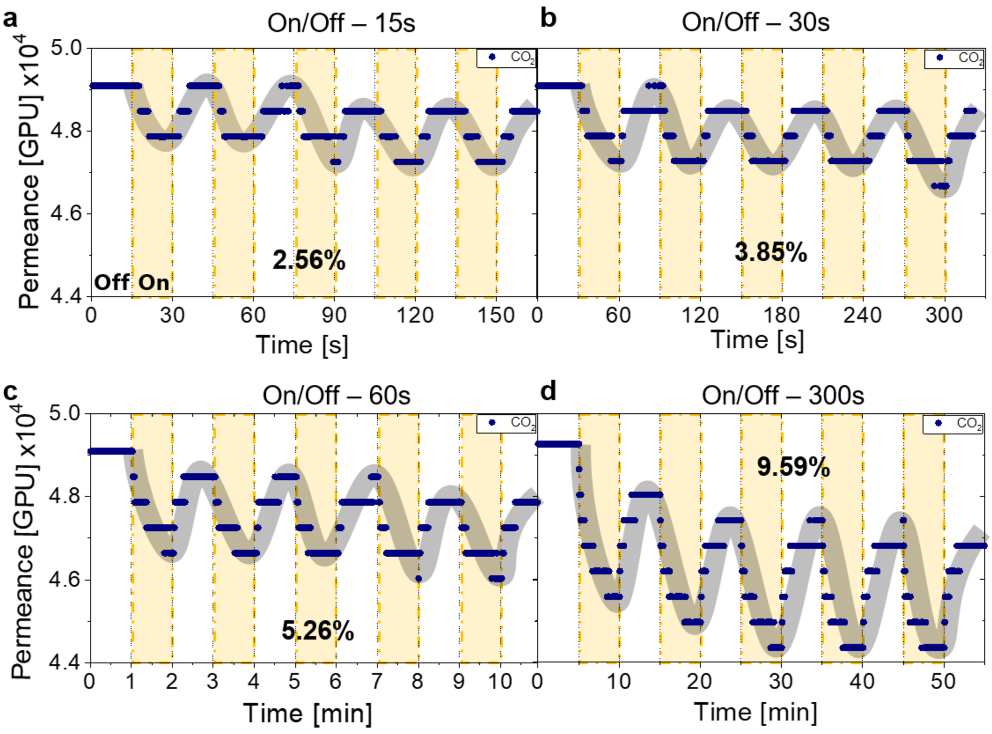

Supplementary Figure 19. The change in CO_2 permeance of **AAOCN-6** sample at 350 mbar transmembrane pressure and maximum light intensity (7.2 W/cm^2) for irradiation time of 15s (a), 30s (b), 60s (c) and 300s (d).

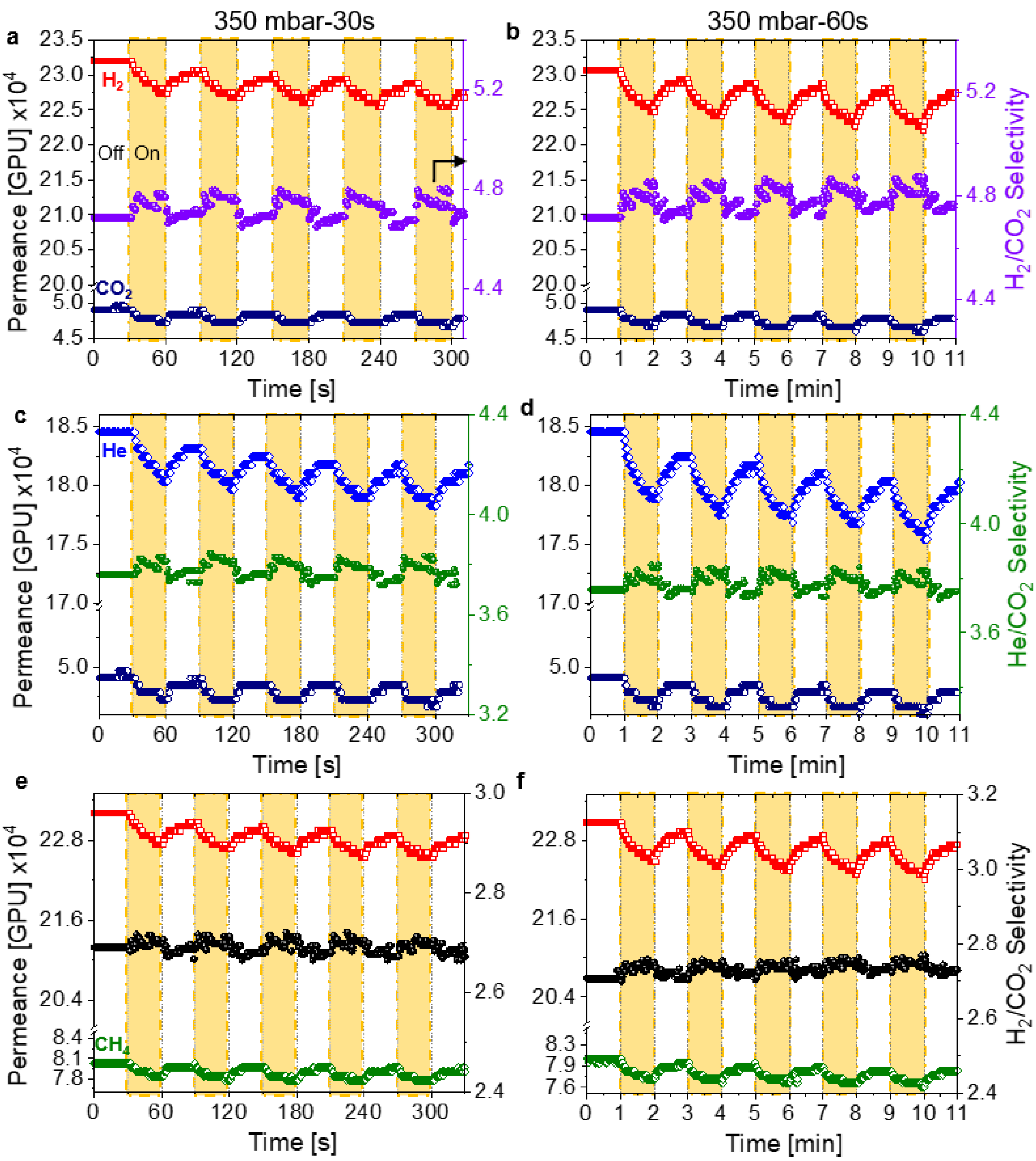

Supplementary Figure 20. The change of gas permeance and H_2/CO_2 selectivity upon irradiation for 30s (a) and 60s (b), He/CO_2 selectivity upon irradiation for 30s (c) and 60s (d), H_2/CH_4 selectivity upon irradiation for 30s (e) and 60s (f) on **AAOCN-6** at 350 mbar.

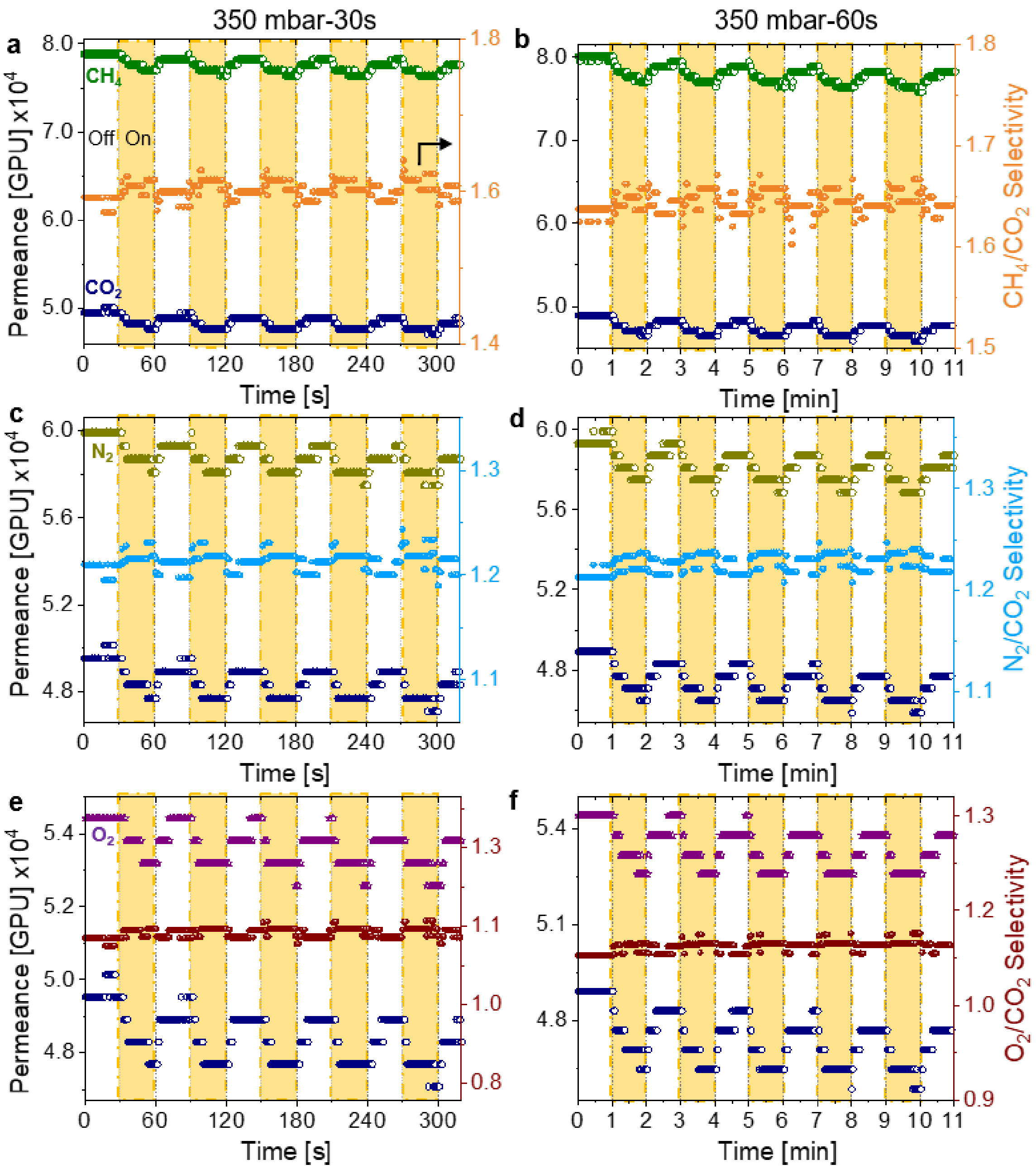

Supplementary Figure 21. The change of gas permeance and CH_4/CO_2 selectivity upon irradiation for 30s (a) and 60s (b), N_2/CO_2 selectivity upon irradiation for 30s (c) and 60s (d), O_2/CO_2 selectivity upon irradiation for 30s (e) and 60s (f) on **AAOCN-6** at 350 mbar.

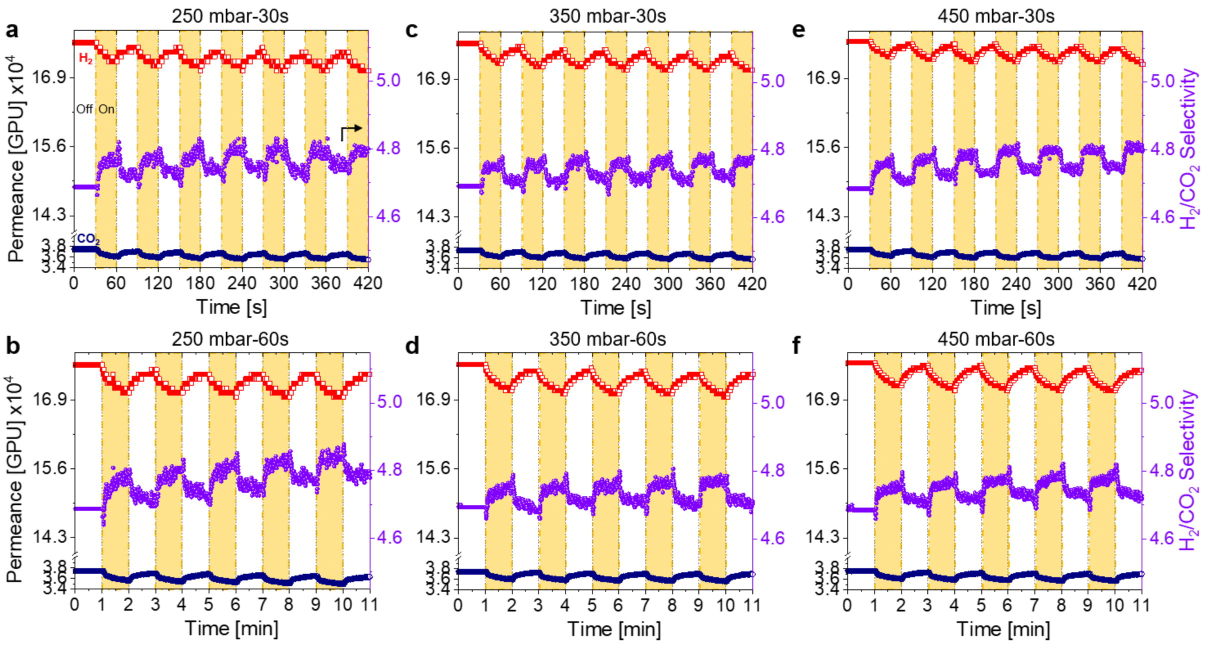

Supplementary Figure 22. The change of gas permeance and H_2/CO_2 selectivity upon irradiation for 30s (a) and 60s (b) at 250 mbar, for 30s (c) and 60s (d) at 350 mbar, for 30s (e) and 60s (f) at 450 mbar transmembrane pressure on **AAOCN-8** sample.

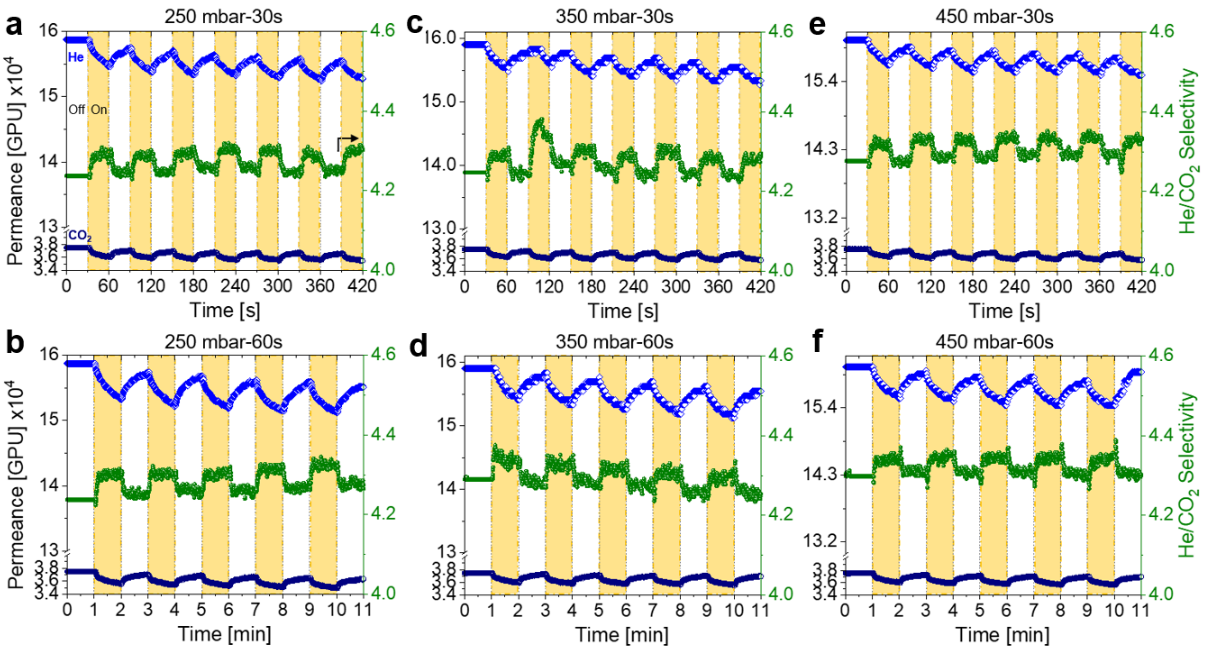

Supplementary Figure 23. The change of gas permeance and He/CO_2 selectivity upon irradiation for 30s (a) and 60s (b) at 250 mbar, for 30s (c) and 60s (d) at 350 mbar, for 30s (e) and 60s (f) at 450 mbar transmembrane pressure on **AAOCN-8** sample.

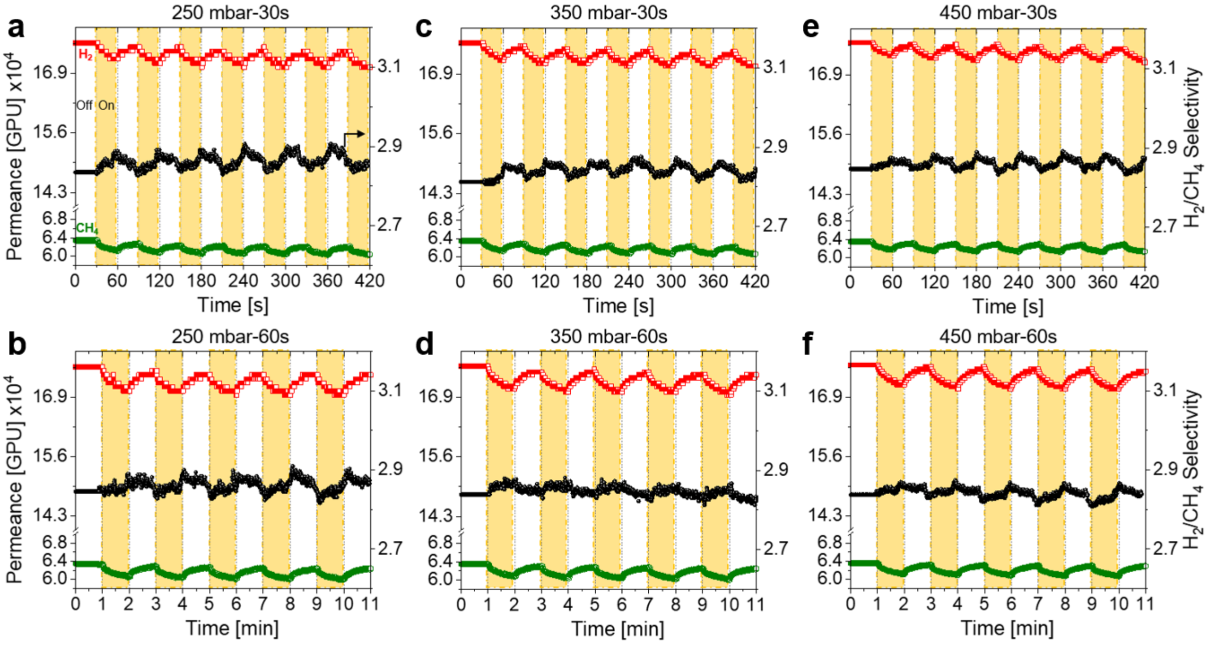

Supplementary Figure 24. The change of gas permeance and H_2/CH_4 selectivity upon irradiation for 30s (a) and 60s (b) at 250 mbar, for 30s (c) and 60s (d) at 350 mbar, for 30s (e) and 60s (f) at 450 mbar transmembrane pressure on **AAOCN-8** sample.

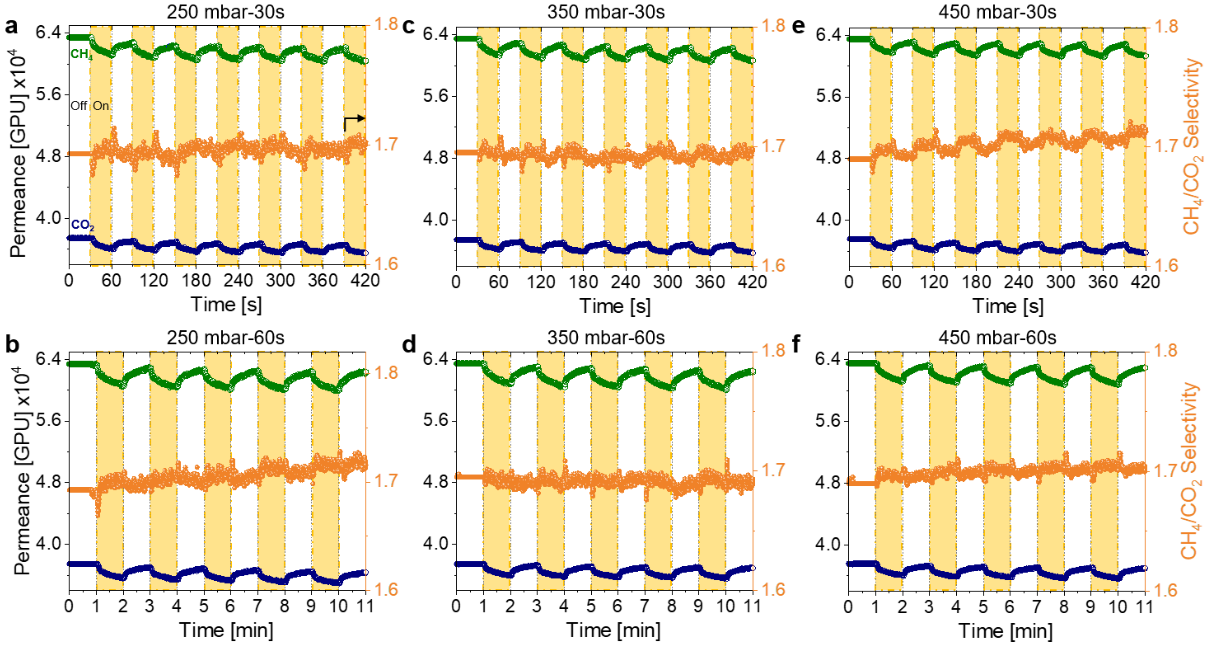

Supplementary Figure 25. The change of gas permeance and CH_4/CO_2 selectivity upon irradiation for 30s (a) and 60s (b) at 250 mbar, for 30s (c) and 60s (d) at 350 mbar, for 30s (e) and 60s (f) at 450 mbar transmembrane pressure on **AAOCN-8** sample.

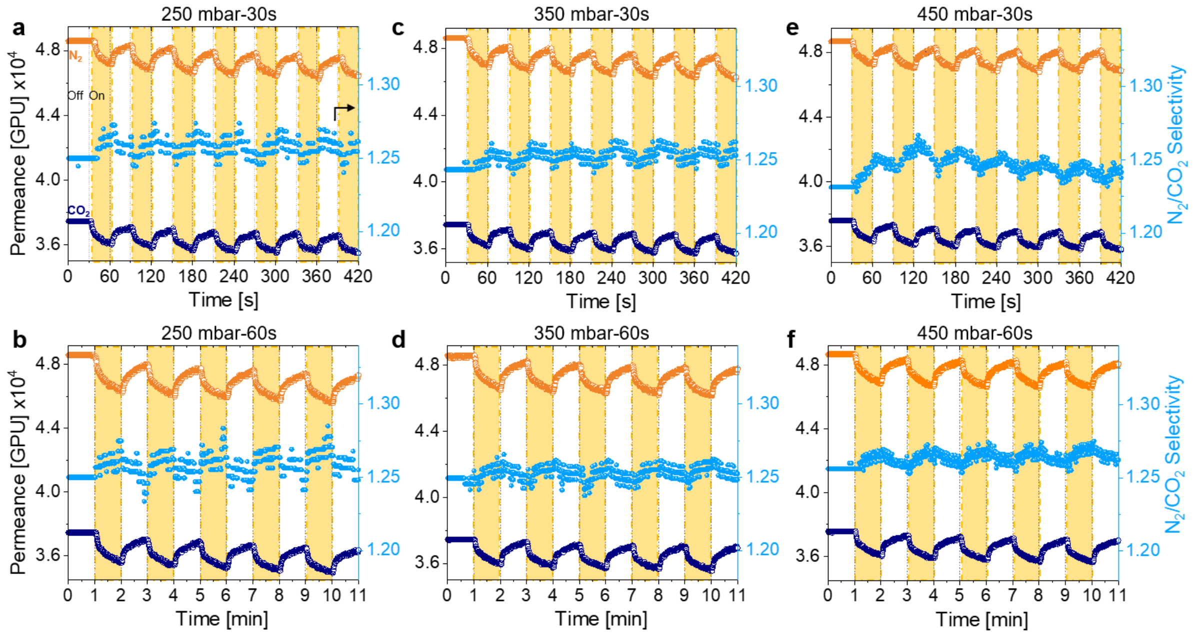

Supplementary Figure 26. The change of gas permeance and N_2/CO_2 selectivity upon irradiation for 30s (a) and 60s (b) at 250 mbar, for 30s (c) and 60s (d) at 350 mbar, for 30s (e) and 60s (f) at 450 mbar transmembrane pressure on **AAOCN-8** sample.

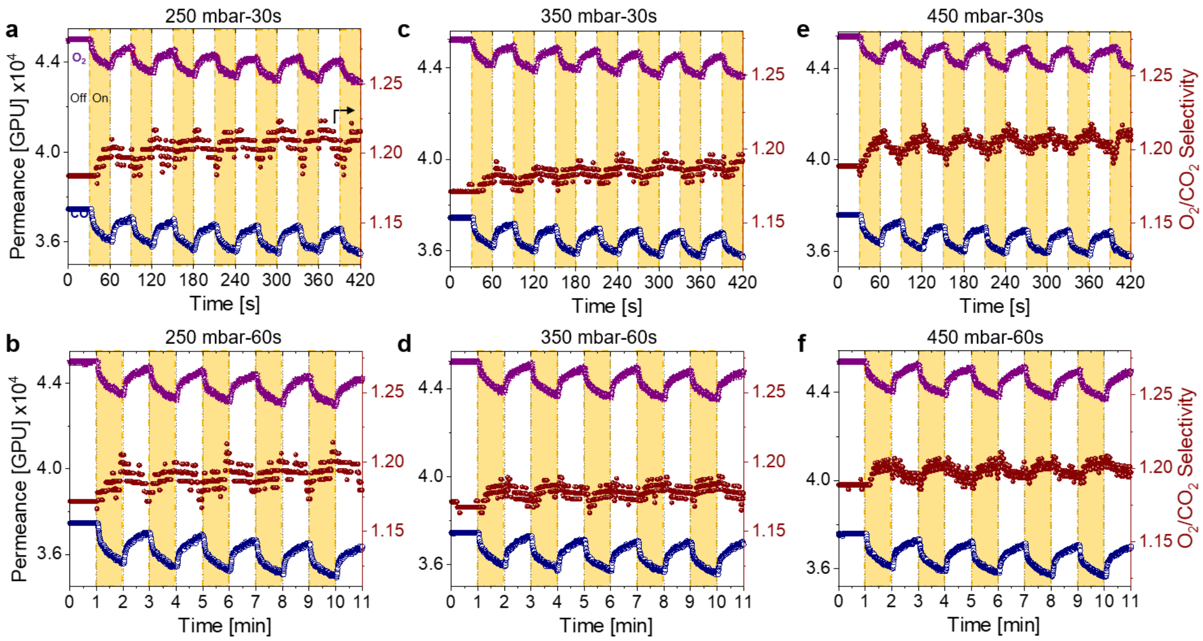

Supplementary Figure 27. The change of gas permeance and O_2/CO_2 selectivity upon irradiation for 30s (a) and 60s (b) at 250 mbar, for 30s (c) and 60s (d) at 350 mbar, for 30s (e) and 60s (f) at 450 mbar transmembrane pressure on **AAOCN-8** sample.

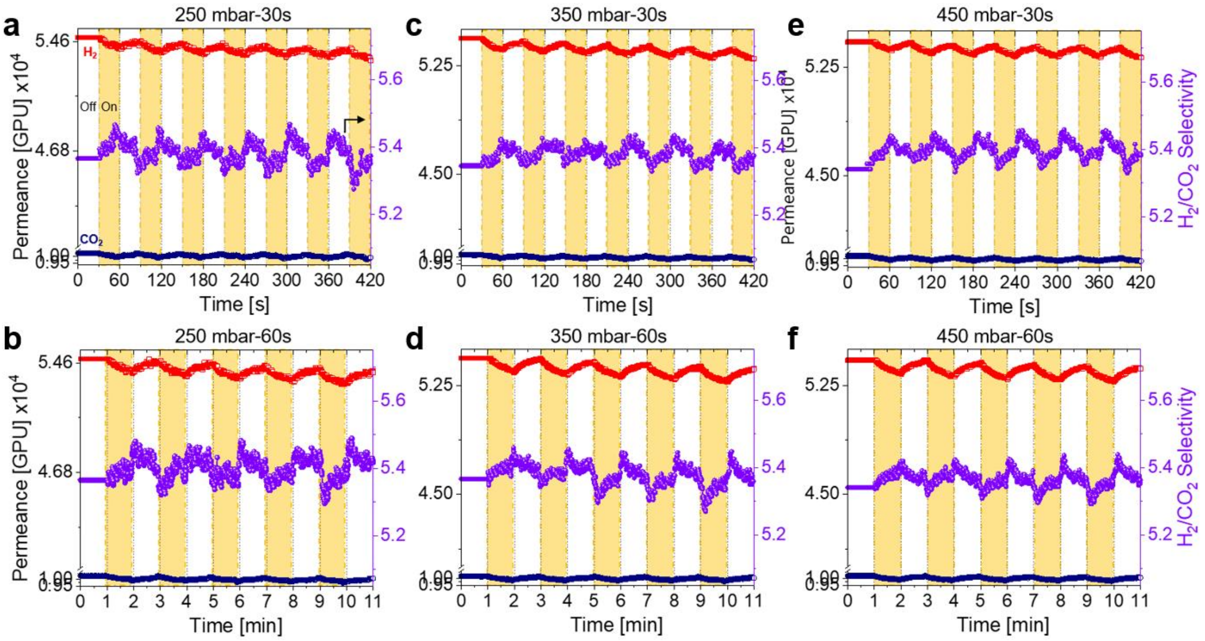

Supplementary Figure 28. The change of gas permeance and H_2/CO_2 selectivity upon irradiation for 30s (a) and 60s (b) at 250 mbar, for 30s (c) and 60s (d) at 350 mbar, for 30s (e) and 60s (f) at 450 mbar transmembrane pressure on **AAOCN-12** sample.

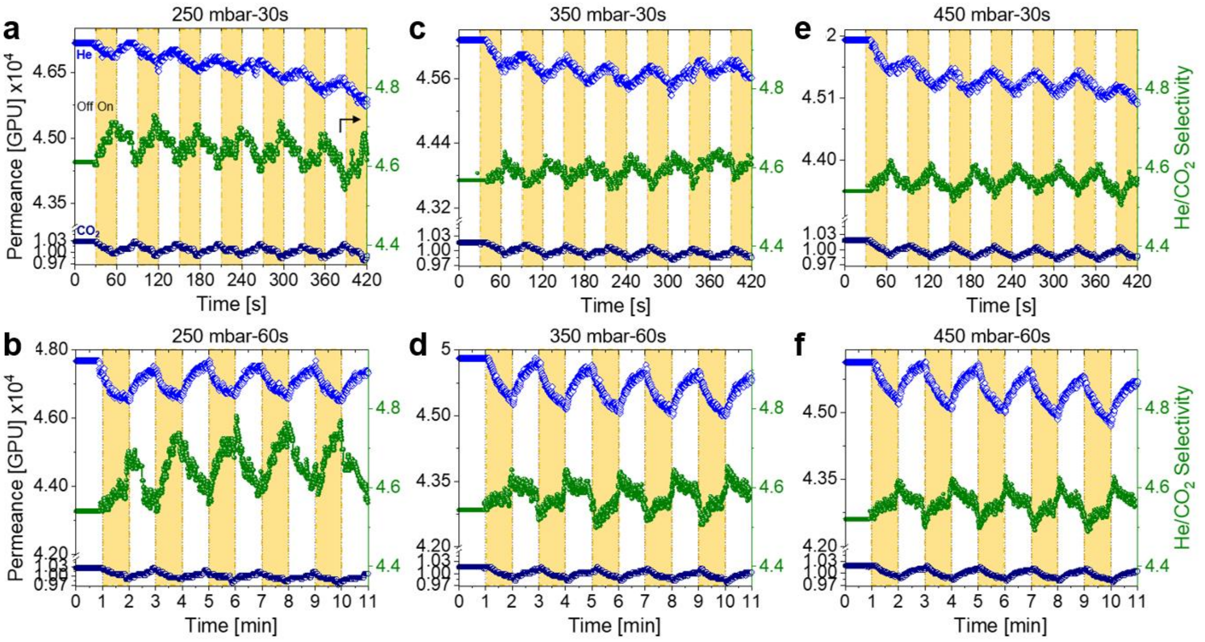

Supplementary Figure 29. The change of gas permeance and He/CO_2 selectivity upon irradiation for 30s (a) and 60s (b) at 250 mbar, for 30s (c) and 60s (d) at 350 mbar, for 30s (e) and 60s (f) at 450 mbar transmembrane pressure on **AAOCN-12** sample.

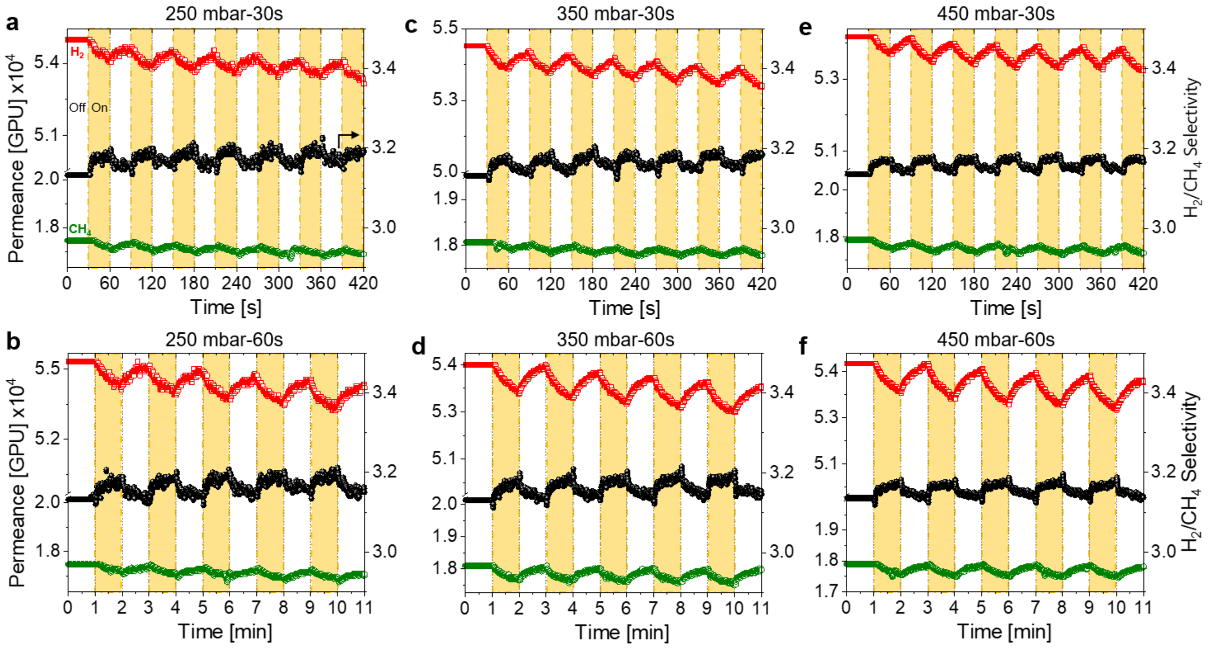

Supplementary Figure 30. The change of gas permeance and H_2/CH_4 selectivity upon irradiation for 30s (a) and 60s (b) at 250 mbar, for 30s (c) and 60s (d) at 350 mbar, for 30s (e) and 60s (f) at 450 mbar transmembrane pressure on **AAOCN-12** sample.

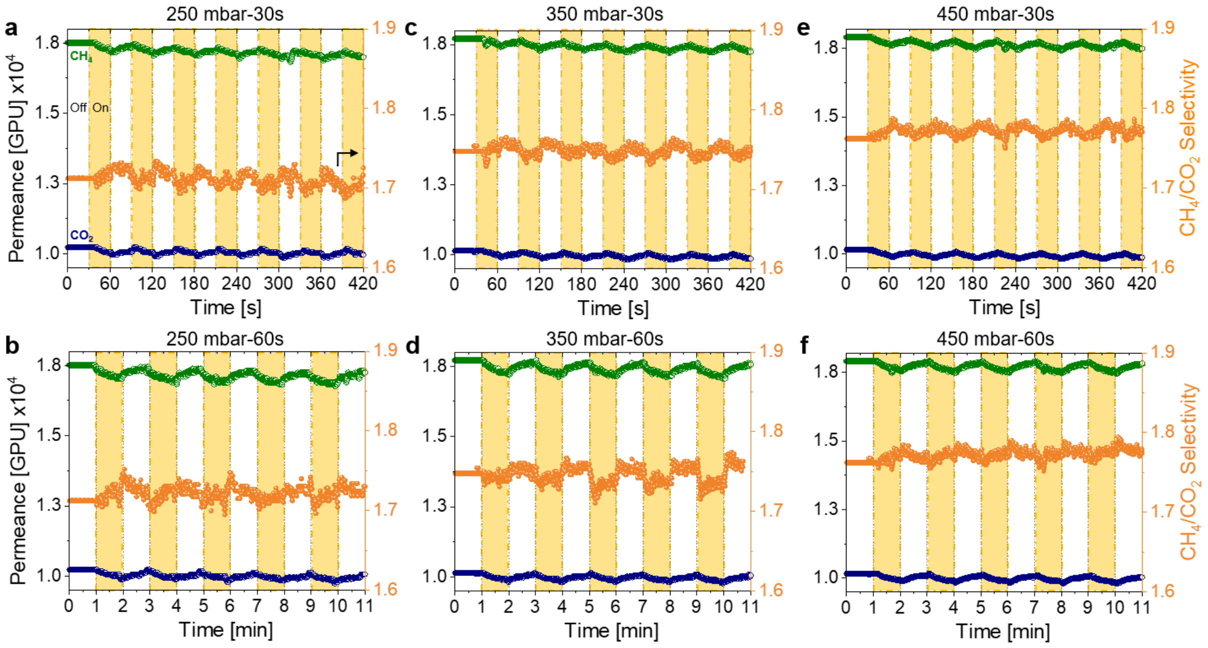

Supplementary Figure 31. The change of gas permeance and CH_4/CO_2 selectivity upon irradiation for 30s (a) and 60s (b) at 250 mbar, for 30s (c) and 60s (d) at 350 mbar, for 30s (e) and 60s (f) at 450 mbar transmembrane pressure on **AAOCN-12** sample.

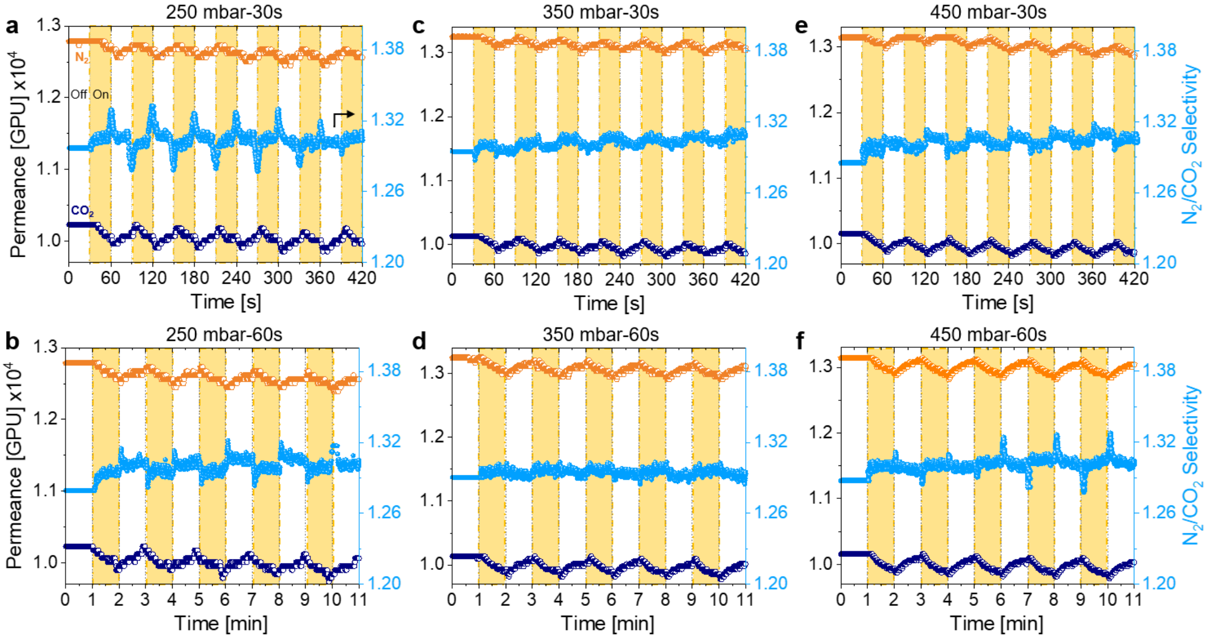

Supplementary Figure 32. The change of gas permeance and N_2/CO_2 selectivity upon irradiation for 30s (a) and 60s (b) at 250 mbar, for 30s (c) and 60s (d) at 350 mbar, for 30s (e) and 60s (f) at 450 mbar transmembrane pressure on **AAOCN-12** sample.

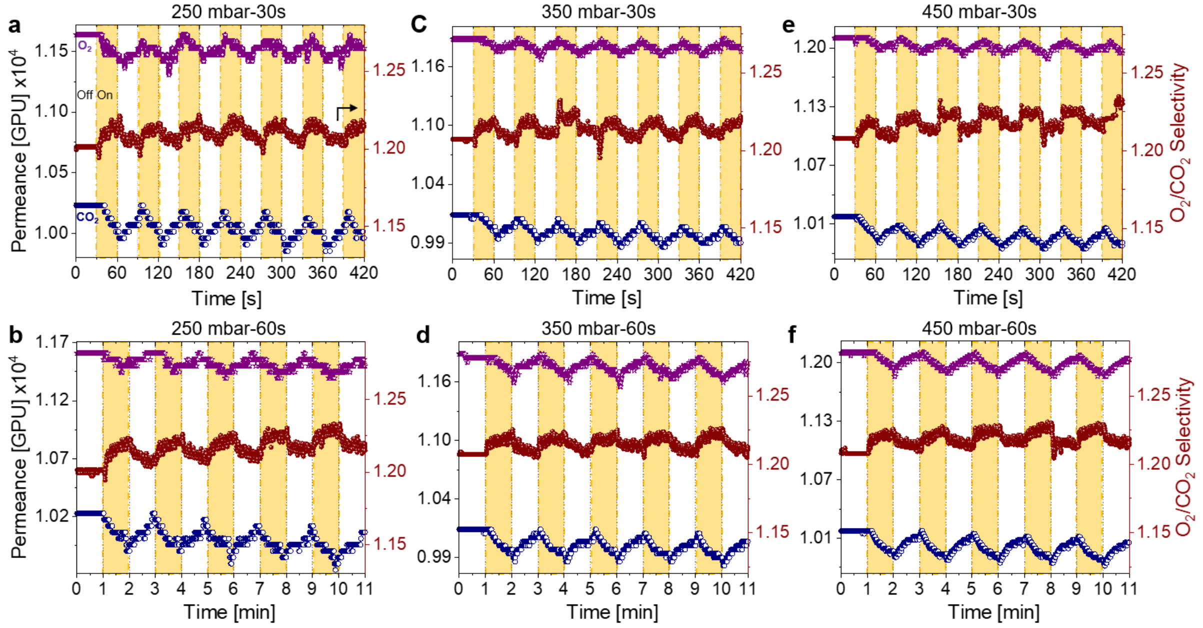

Supplementary Figure 33. The change of gas permeance and O_2/CO_2 selectivity upon irradiation for 30s (a) and 60s (b) at 250 mbar, for 30s (c) and 60s (d) at 350 mbar, for 30s (e) and 60s (f) at 450 mbar transmembrane pressure on **AAOCN-12** sample.

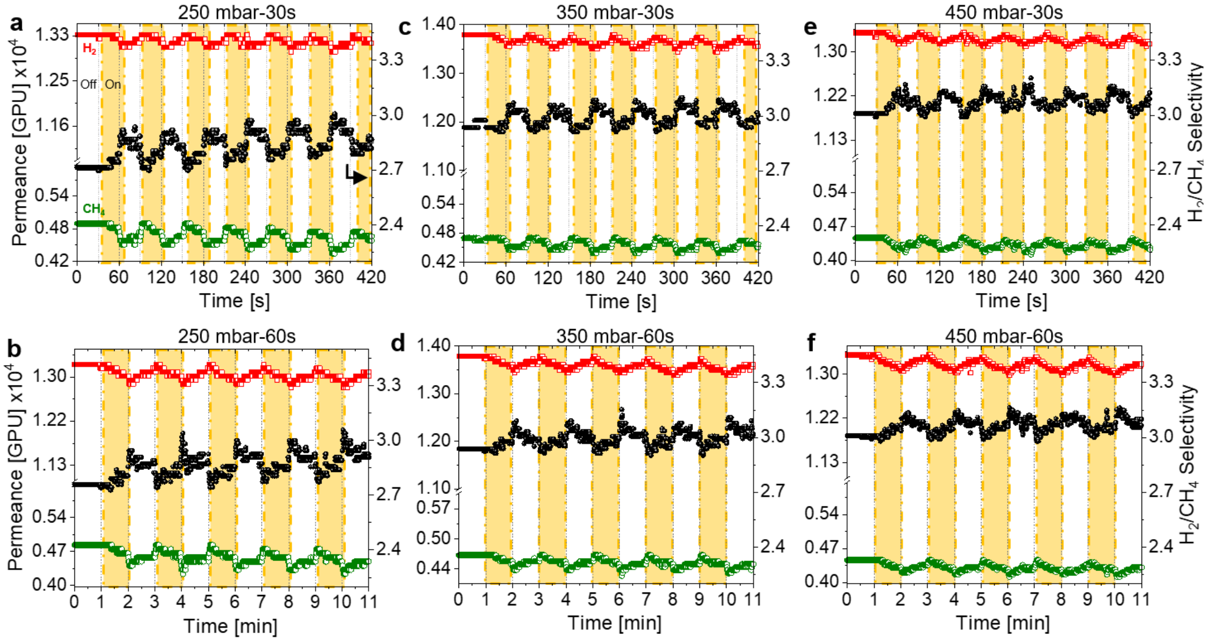

Supplementary Figure 34. The change of gas permeance and H_2/CH_4 selectivity upon irradiation for 30s (a) and 60s (b) at 250 mbar, for 30s (c) and 60s (d) at 350 mbar, for 30s (e) and 60s (f) at 450 mbar transmembrane pressure on **AAOCN-16** sample.

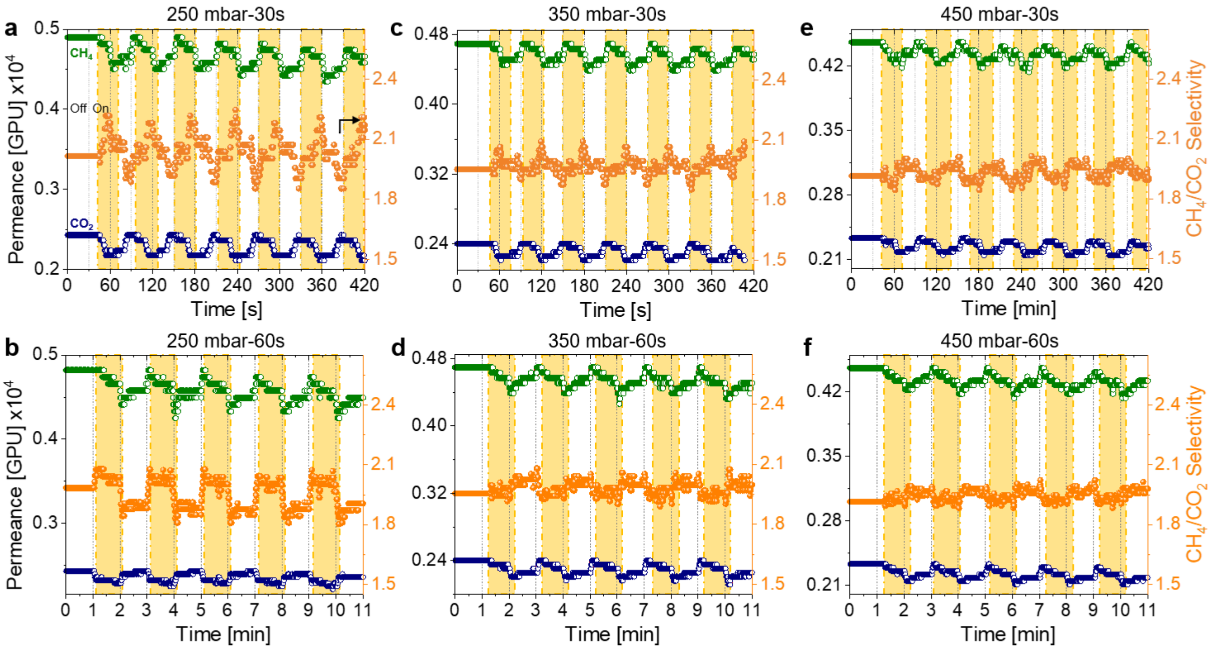

Supplementary Figure 35. The change of gas permeance and CH_4/CO_2 selectivity upon irradiation for 30s (a) and 60s (b) at 250 mbar, for 30s (c) and 60s (d) at 350 mbar, for 30s (e) and 60s (f) at 450 mbar transmembrane pressure on **AAOCN-16** sample.

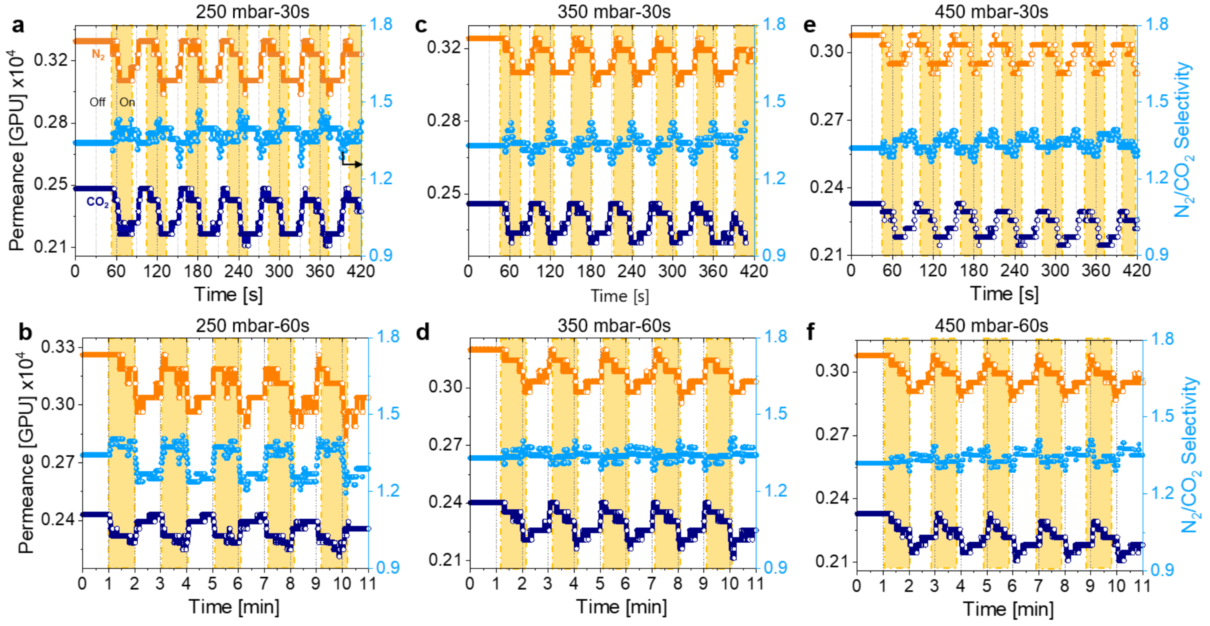

Supplementary Figure 36. The change of gas permeance and N_2/CO_2 selectivity upon irradiation for 30s (a) and 60s (b) at 250 mbar, for 30s (c) and 60s (d) at 350 mbar, for 30s (e) and 60s (f) at 450 mbar transmembrane pressure on **AAOCN-16** sample.

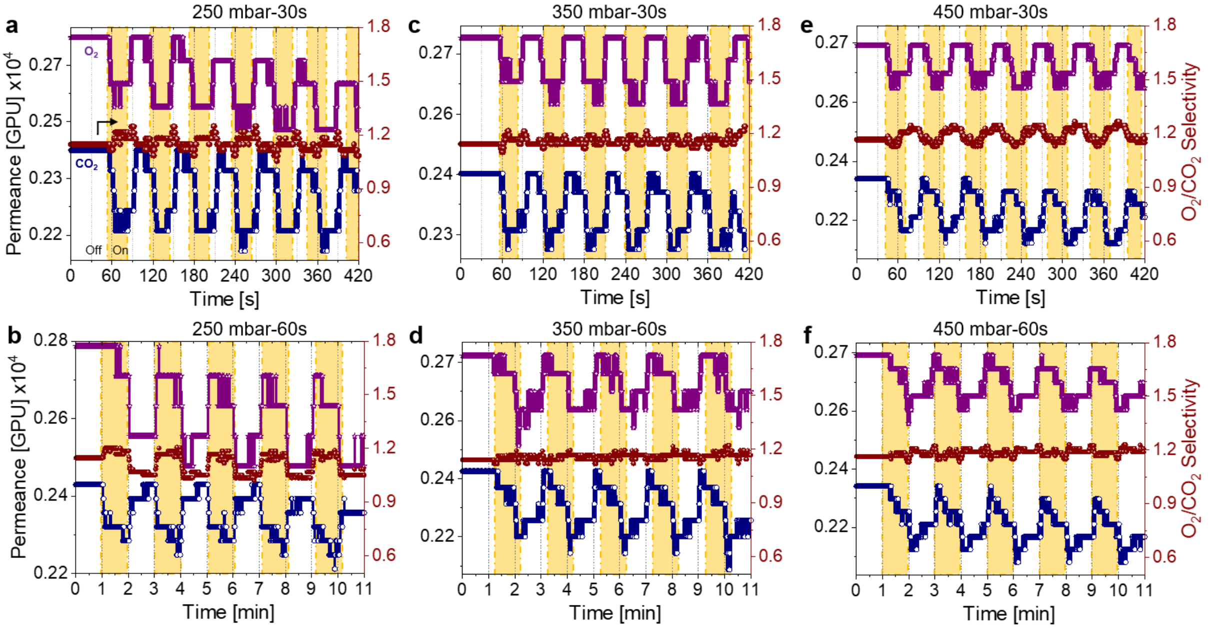

Supplementary Figure 37. The change of gas permeance and O_2/CO_2 selectivity upon irradiation for 30s (a) and 60s (b) at 250 mbar, for 30s (c) and 60s (d) at 350 mbar, for 30s (e) and 60s (f) at 450 mbar transmembrane pressure on **AAOCN-16** sample.

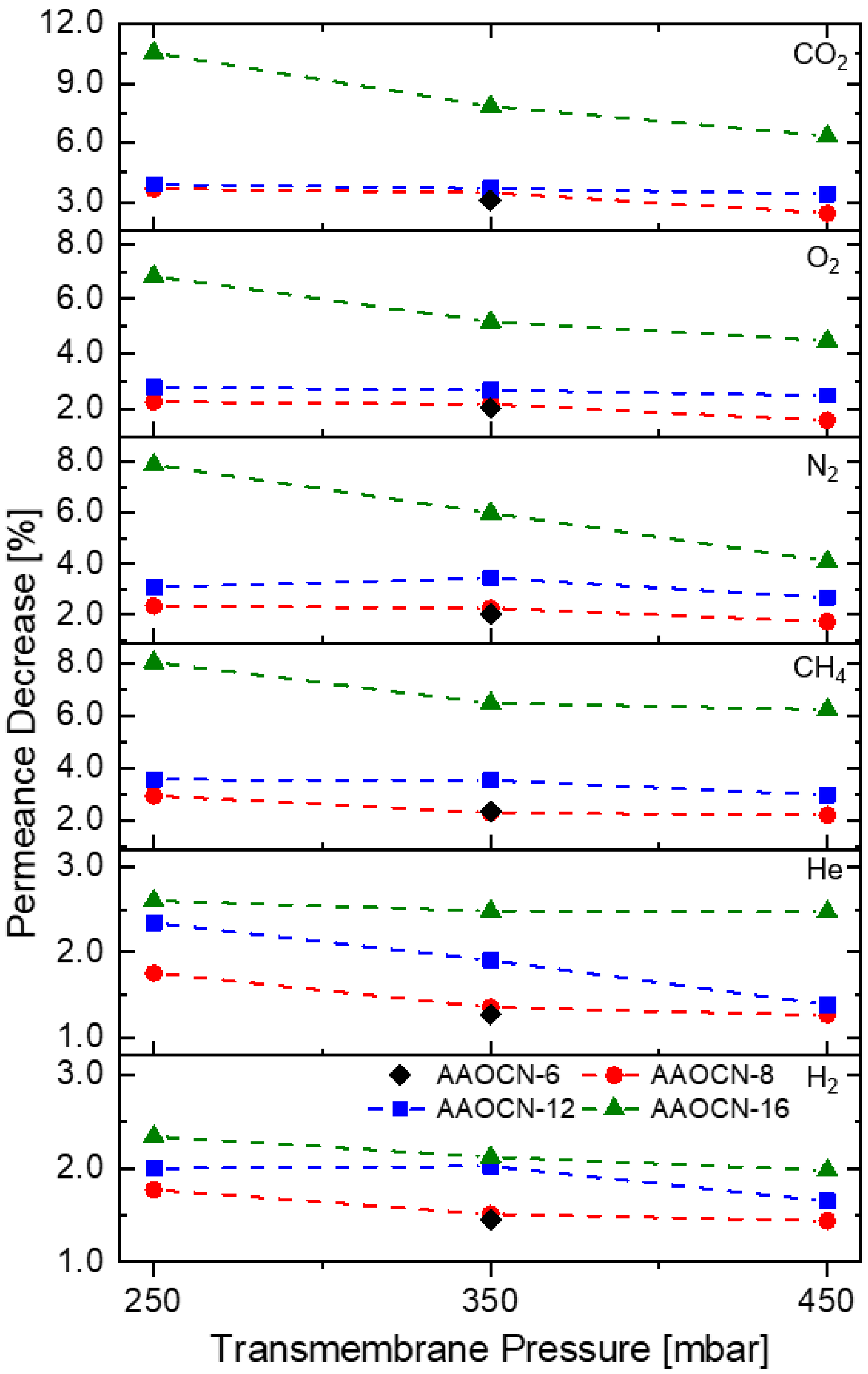

Supplementary Figure 38. The decrease in the gas permeance during irradiation for 30s at maximum light intensity (7.2 W/cm^2) vs transmembrane pressure for **AAOCN-X** samples.

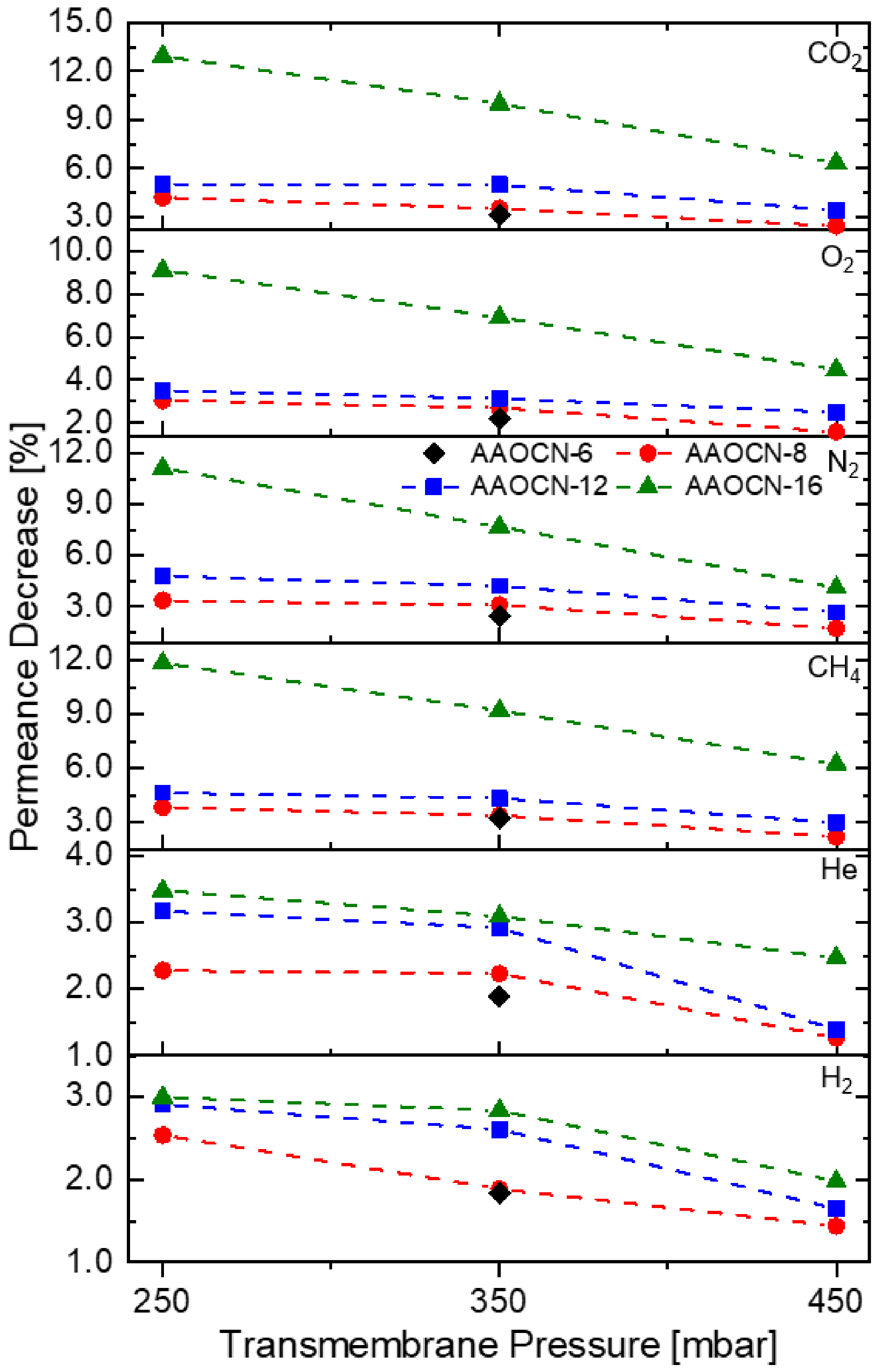

Supplementary Figure 39. The decrease in gas permeance during irradiation for 60s at maximum light intensity (7.2 W/cm^2) vs transmembrane pressure for **AAOCN-X** samples.

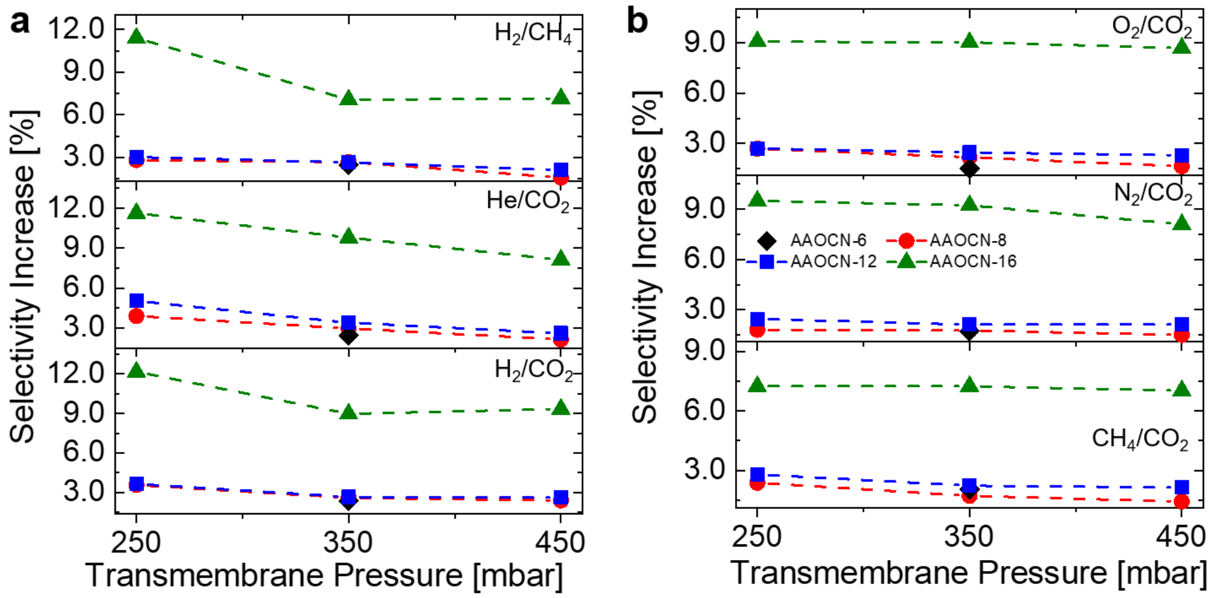

Supplementary Figure 40. The increase in the gas selectivity during irradiation for 30s at maximum light intensity (7.2 W/cm^2) vs transmembrane pressure for H_2/CO_2, He/CO_2, H_2/CH_4 (a) and for CH_4/CO_2, N_2/CO_2, O_2/CO_2 (b) on **AAOCN-X** samples.

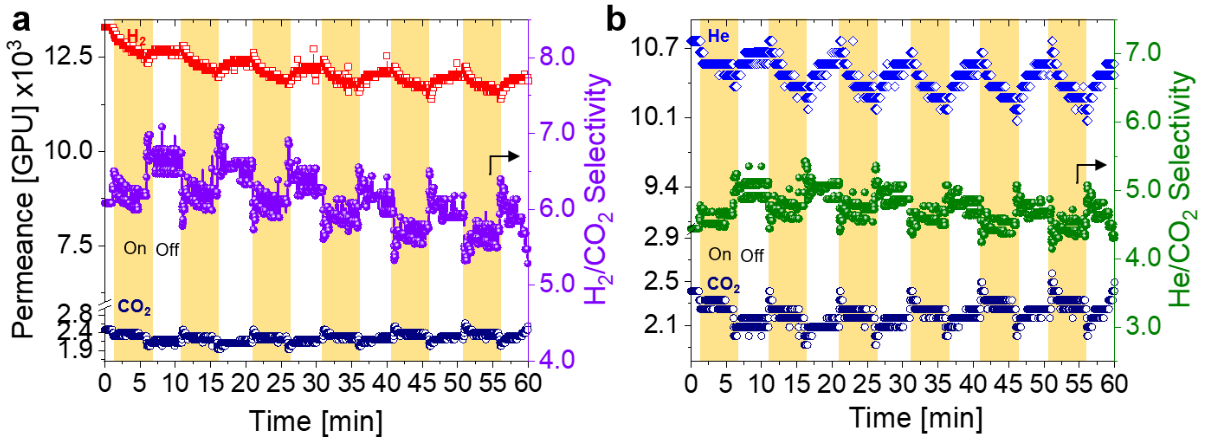

Supplementary Figure 41. The change of gas permeance and H_2/CO_2 selectivity (a) and He/CO_2 selectivity (b) upon irradiation for 300s at 250 mbar transmembrane pressure on **AAOCN-16** sample.

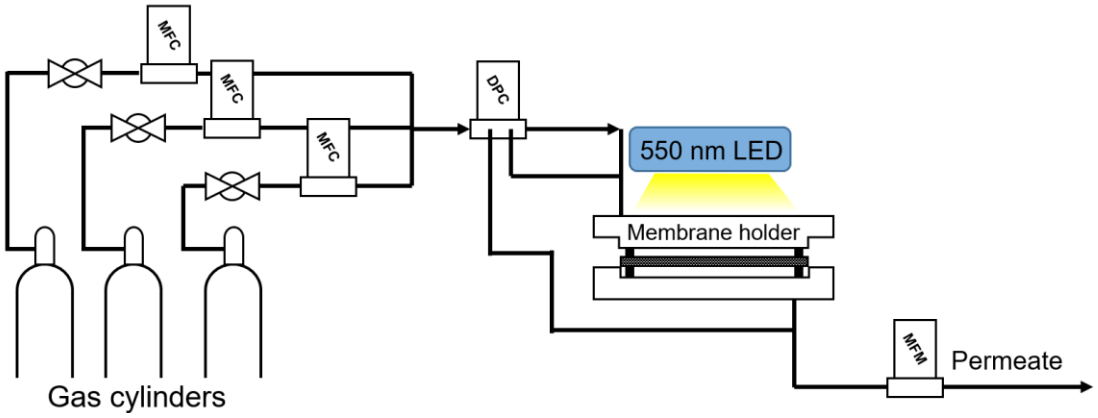

Supplementary Figure 42. Schematic representation of the gas permeation measurement setup: MFC-mass flow controller, DPC-differential pressure controller, MFM-mass flow meter. Membranes were held between two *O*-rings to avoid any leaks. DPC has a 4-way connection and controls the transmembrane pressure by controlling pressure difference between feed and permeate sides. A 550 nm LED light source was used for irradiation. Arrows indicate the gas flow direction.

Supplementary Tables

Supplementary Table S1. Deposition parameters for preparation of AAOCN-X membranes.

CN x g	Step 1	Step 2	Step 3	Step 4	Step 5	Step 6
Up stream (ºC)	0	0	300	300	0	0
Down stream (ºC)	0	550	550	550	550	0
Heater time (min)	10	40	30	60	30	2
N_2 gas set (sccm)	500	50	50	50	50	0
Pressure set (torr)	10	10	10	10	10	10

*Amount of precursor (melamine) x = 6 g, 8 g, 12 g, 16 g and 20 g.

Supplementary Table S2. Experimental polarizabilities of the gases.

Gas	***Polarizability (Å³)*** [1]
H_2	0.787
He	0.208
CH_4	2.448
N_2	1.710
O_2	1.562
CO_2	2.507

Supplementary Table S3. Binding energy of gases with pCN.

	Binding energy (kJ/mol)	
	pCN	pCN (+1)
CO_2	-32.9	-35.5
CH_4	-20.1	-22.9
H_2	-10.8	-10.9
He	-3.2	-3.8

.

Supplementary Table S4. The performance comparison of our pCN-based light-switchable membranes with previously reported stimuli responsive gas separation membranes.

**Membrane/Material**	**Switching mechanism**	**Stimuli /Controllability**	**Response time**	**Selectivity change**	**Gas permeance (GPU)***	**Reference**
Zeolitic imidazolate framework- ZIF-8 on AAO support	Switching of the ZIF-8 into polar polymorphs	E-field: 500 V/mm<br>Not controllable	30-60 min	H_2/CO_2: decreased from ~4 to ~1.7 (-57.5%)<br><br>H_2/CH_4: decreased from ~11 to ~2 (-81.8%)<br><br>CO_2/CH_4: decreased from ~2.1 to ~1.5 (-28.6%)<br><br>Propane/Propene: decreased from ~7.3 to ~5.8 (-20.5%)	H_2: ~1791<br><br>CO_2: ~340<br><br>Propane: ~12	[4]
Azobenzene guest Molecules on a Ultrathin UiO-67 Membrane	Transformation of Azobenzene from *cis* to *trans* configuration	Light: 365 nm for "*cis*" and 455 nm for "*trans*" state<br>Irradiation time	~120 min from *cis* to *trans* and 106 for vice-versa	H_2/CO_2: decreased from ~14.7 to ~10.1 (-31.2%)	H_2: 1522	[5]
SURMOFs	Transformation of Azobenzene from *cis* to *trans* configuration	Light: 365 nm for "*cis*" and 455 nm for "*trans*" state<br>Irradiation time	~10-30 min	H_2/CO_2: increased from ~3 to ~7.9 (163.3%)<br><br>N_2/CO_2: increased from ~5.5 to ~8.2 (49.1%)	H_2: ~746<br><br>N_2: ~2668	[6]
SURMOFs	Transformation of Azobenzene from *cis* to *trans* configuration	Light: 400 nm for "*cis*" and 530 nm for "*trans*" state	~10-30 min	H_2/C_2H_4: decreased from ~8.2 to ~6.6 (-19.5%)	H_2: ~2447	[7]

		Irradiation time		H_2/C_3H_6: decreased from<br>~12.6 to ~8.8 (-30.2%)		
Poly(methylmethacrylate)-Azobenzene Membrane	Transformation of Azobenzene from *cis* to *trans* configuration	Light: 360 nm for "*cis*" and 440 nm for "*trans*" state<br>Irradiation time	~10 min	H_2/SF_6: increased from ~25 to ~100 (300%)<br>H_2/CH_4: increased from ~10 to ~17 (70%)	H_2: ~0.74	[8]
azo-PI[g-h]<br>azo-PI[g-h]_UV	Transformation of Azobenzene from *cis* to *trans* configuration	Light: 405 nm for "*cis*" state<br>Irradiation time	~5 min	O_2/N_2: decreased from 10.0 to 8.6 (-16.3%)<br>He/N_2: increased from 388 to 399 (2.8%)<br>CO_2/N_2: decreased from 37.1 to 29.0 (-27.9%)	O_2: ~0.0017<br><br>He: ~0.067<br><br>CO_2: ~0.064	[9]
azo-coPI[g-h]<br>azo-coPI[g-h]_UV				O_2/N_2: decreased from 6.9 to 6.3 (-9.5%)<br>He/N_2: decreased from 160 to 155 (-3.2%)<br>CO_2/N_2: decreased from 26.7 to 21.9 (-21.9%)	O_2: ~0.0085<br><br>He: ~0.20<br><br>CO_2: ~0.033	
Na–X–AZB membrane	Transformation of Azobenzene from *cis* to *trans* configuration	Light: 360 nm for "*cis*" and 440 nm for "*trans*" state<br>Irradiation time	~5 min	N_2/CO_2: decreased from<br>49 to 32 (-34.7%)	N_2: ~0.003	[10]
trans and *cis*-PI-1	Transformation of Azobenzene from *cis* to *trans* configuration	Light: 405 nm for "*cis*" state<br>Irradiation time	~3 min	He/CO_2: increased from 3.47 to 3.71 (6.8%)<br>O_2/N_2: increased from 6.94 to 7.25 (4.5%)<br>CO_2/N_2: decreased from 29.61 to 29.17 (1.5%)	He: ~0.19<br>O_2: ~0.013<br>CO_2: ~0.052	[11]
Matrimid-JUC-62 and Matrimid-PCN-250	Transformation of Azobenzene from *cis* to *trans* configuration	Light: 360 nm for "*cis*" and 440 nm for "*trans*" state	~83 min	CO_2 permeance decreased:<br>5wt% JUC-62-matrimid: from 0.122 to 0.114 (-6.4%)<br>10wt% JUC-62-matrimid: from 0.292 to 0.278 (-4.9%)		[12]

mixed matrix membranes		Not controllable		15wt% JUC-62-matrimid: from 0.339 to 0.31 (-8.5%)<br>5wt% PCN-250-matrimid: from 0.206 to 0.193 (-5.9%)<br>10wt% PCN-250-matrimid: from 0.331 to 0.317 (-5.0%)		
In-situ grown pCN on AAO	Redistribution of the charges on pCN	Light: 550 nm<br>Irradiation time<br>Irradiation power	~1 s**	H_2/CO_2: increased from<br>5.8 to 7.1 (22.4%) at 5 min of irradiation<br><br>He/CO_2: increased from<br>4.4 to 5.4 (22.6%) at 5 min of irradiation<br><br>H_2/CH_4: increased from<br>~2.8 to ~3.1 (10.7%) at irradiation of 1 minutes	H_2: 13298<br><br>He: 10792	**This work**

* 1 GPU = 3.35×10^{-10} mol s^{-1} m^{-2} Pa^{-1}

** The response time is the time required for the change to be observed. The amount of change is directly proportional with the irradiation duration.

Supplementary References

1 Olney, T. N., Cann, N. M., Cooper, G. & Brion, C. E. Absolute scale determination for photoabsorption spectra and the calculation of molecular properties using dipole sum-rules. *Chem. Phys.* **223**, 59-98, doi:https://doi.org/10.1016/S0301-0104(97)00145-6 (1997).

2 Weh, K. *et al.* Modification of the Transport Properties of a Polymethacrylate-Azobenzene Membrane by Photochemical Switching. *Chem. Eng. Tech.* **21**, 408-412, doi:https://doi.org/10.1002/(SICI)1521-4125(199805)21:5<408::AID-CEAT408>3.0.CO;2-L (1998).

3 Prasetya, N., Teck, A. A. & Ladewig, B. P. Matrimid-JUC-62 and Matrimid-PCN-250 mixed matrix membranes displaying light-responsive gas separation and beneficial ageing characteristics for CO_2/N_2 separation. *Sci. Rep.* **8**, 2944, doi:10.1038/s41598-018-21263-7 (2018).

4 Knebel, A. *et al.* Defibrillation of soft porous metal-organic frameworks with electric fields. *Science* **358**, 347-351, doi:doi:10.1126/science.aal2456 (2017).

5 Knebel, A. *et al.* Azobenzene Guest Molecules as Light-Switchable CO2 Valves in an Ultrathin UiO-67 Membrane. *Chem. Mater.* **29**, 3111-3117, doi:10.1021/acs.chemmater.7b00147 (2017).

6 Wang, Z. *et al.* Tunable molecular separation by nanoporous membranes. *Nature Communications* **7**, 13872, doi:10.1038/ncomms13872 (2016).

7 Müller, K. *et al.* Switching Thin Films of Azobenzene-Containing Metal–Organic Frameworks with Visible Light. *Chemistry – A European Journal* **23**, 5434-5438, doi:https://doi.org/10.1002/chem.201700989 (2017).

8 Weh, K. *et al.* Modification of the Transport Properties of a Polymethacrylate-Azobenzene Membrane by Photochemical Switching. *Chemical Engineering & Technology* **21**, 408-412, doi:https://doi.org/10.1002/(SICI)1521-4125(199805)21:5<408::AID-CEAT408>3.0.CO;2-L (1998).

9 Nocoń-Szmajda, K., Jankowski, A., Wolińska-Grabczyk, A. & Konieczkowska, J. Guest-host and functionalized side-chain azopolyimide membranes for controlled gas separation. *Polymer* **229**, 124012, doi:https://doi.org/10.1016/j.polymer.2021.124012 (2021).

10 Weh, K., Noack, M., Hoffmann, K., Schröder, K. P. & Caro, J. Change of gas permeation by photoinduced switching of zeolite-azobenzene membranes of type MFI and FAU. *Microporous Mesoporous Mater.* **54**, 15-26, doi:https://doi.org/10.1016/S1387-1811(02)00331-1 (2002).

11 Bujak, K. *et al.* Azopolymers with imide structures as light-switchable membranes in controlled gas separation. *European Polymer Journal* **118**, 186-194, doi:https://doi.org/10.1016/j.eurpolymj.2019.05.051 (2019).

12 Prasetya, N., Teck, A. A. & Ladewig, B. P. Matrimid-JUC-62 and Matrimid-PCN-250 mixed matrix membranes displaying light-responsive gas separation and beneficial ageing characteristics for CO_2/N_2 separation. *Scientific Reports* **8**, 2944, doi:10.1038/s41598-018-21263-7 (2018).
